# Mild temperature photothermal assisted anti-bacterial and anti-inflammatory nanosystem for synergistic treatment of post-cataract surgery endophthalmitis

**DOI:** 10.7150/thno.46895

**Published:** 2020-07-09

**Authors:** Yang Ye, Jian He, Yue Qiao, Yuchen Qi, Hongbo Zhang, Hélder A. Santos, Danni Zhong, Wanlin Li, Shiyuan Hua, Wei Wang, Andrzej Grzybowski, Ke Yao, Min Zhou

**Affiliations:** 1 Eye Center, The Second Affiliated Hospital, Zhejiang University School of Medicine, Hangzhou 310009, China.; 2 Institute of Translational Medicine, Zhejiang University, Hangzhou, 310009, China.; 3 Zhejiang Provincial Key Lab of Ophthalmology, Hangzhou, 310009, China.; 4 Department of Pharmaceutical Science, Åbo Akademi University; Turku Bioscience Center, University of Turku and Åbo Akademi University, FI-20520, Finland.; 5 Drug Research Program, Division of Pharmaceutical Chemistry and Technology, Faculty of Pharmacy, University of Helsinki, FI-00014, Finland.; 6 Key Laboratory of Cancer Prevention and Intervention, National Ministry of Education, Zhejiang University, Hangzhou, 310009, China.; 7 State Key Laboratory of Modern Optical Instrumentations, Zhejiang University, Hangzhou, 310058, China.; 8 Department of Ophthalmology, University of Warmia and Mazury, Olsztyn, Poland.; 9 Helsinki Institute of Life Science (HiLIFE), University of Helsinki, FI-00014 Helsinki, Finland.

**Keywords:** endophthalmitis, antibacterial and anti-inflammation effect, mild photothermal therapy, nanoparticles, post-cataract Surgery

## Abstract

**Rationale:** Endophthalmitis, which is one of the severest complications of cataract surgeries, can seriously threaten vision and even lead to irreversible blindness owing to its complicated microenvironment, including both local bacterial infection and severe inflammation. It is urgent to develop a comprehensive treatment for both anti-bacterial and anti-inflammatory effects.

**Methods:** Herein, we developed AuAgCu_2_O-bromfenac sodium nanoparticles (AuAgCu_2_O-BS NPs), which was designed to combine anti-bacterial and anti-inflammatory effects for integrated therapy of endophthalmitis after cataract surgery. The AuAgCu_2_O-BS NPs could eradicate methicillin-resistant *Staphylococcus aureus* (MRSA) bacterial strain relied on their photodynamic effects and the release of metal ions (Ag^+^ and Cu^+^) by the hollow AuAgCu_2_O nanostructures mediated mild photothermal effects. The anti-inflammatory drug, bromfenac sodium, released from the nanoparticles were able to significantly reduce the local inflammation of the endophthalmitis and promote tissue rehabilitation. *In vivo* bacterial elimination and anti-inflammation were confirmed by a postcataract endophthalmitis rabbit model.

**Results:** Excellent antibacterial ability of AuAgCu_2_O-BS NPs was verified both *in vitro* and *in vivo*. Ophthalmological clinical observation and pathologic histology analysis showed prominent treatment of inflammatory reaction. Importantly, the mild temperature photothermal effect not only promoted the release of metal ions and bromfenac sodium but also avoided the thermal damage of the surrounding tissues, which was more suitable for the practical application of ophthalmology due to the complex structure of the eyeball. Moreover, superior biocompatibility was approved by the preliminary toxicity investigations, including low cytotoxicity, negligible damage to major organs, and stable intraocular pressure.

**Conclusions:** Our studies of nanosystem provide a promising synergic therapeutic strategy for postcataract endophthalmitis treatment with favorable prognosis and promise in clinical translations.

## Introduction

With the longevity of the population, morbidity and surgery of cataract have increased obviously 1. The World Health Organization (WHO) reported that the number of visual impairment is about 285 million worldwide, and about 94 million people are caused by cataract 2. Endophthalmitis, which is one of the severest complications of cataract surgeries, draws more attention to the public due to the large quantity and even worse in developing countries 3, 4. The reported rate of post-operative endophthalmitis varies between a range of 0.04%-0.2% 1. As an infectious disease, endophthalmitis is usually caused after pathogens including bacteria and fungus spread into eyes through operation and trauma while mostly caused by bacteria mainly including coagulase-negative *staphylococci*, *Staphylococcus aureus* and *Streptococcus* 5-8. Severe infection can easily lead to inflammation aggravation, hypopyon, ocular penetrating, and even blindness 9, 10. In the clinic, the common treatment is an intraocular injection of antibiotics, however, always along with vitrectomy 11 and even evisceration 12. With the overuse and misuse of antibiotics and the prevalence of multidrug-resistant bacteria, the treatment effect of single antibiotic therapy is impeded 13, 14. Therefore, it is urgent to develop a timely and effective method to treat intraocular infection.

Recently, several antibacterial nanomaterials were reported due to their superiority of antibacterial ability with drug-resistant and drug-loading capacity with sustained release 15-17. The release of antimicrobial agents such as metallic ions is a valid method 18, 19. Because of the multiple antibacterial mechanisms aiming at the structure and physiological processes of bacteria, the NPs based antibacterial agents exhibit sustained broad-spectrum antibacterial effects 20. For example, Ag ion and Ag-based compounds that can influence the cell membrane and exhibit broad-spectrum antimicrobial effect 21-24. Besides, Cu ion can serve as an antimicrobial agent while its wound-healing promotion effect is approved 25, 26.

As a transparent optical organ of eyeball, phototherapy has broad prospects on the ophthalmic disease. Phototherapy, including photothermal therapy (PTT) and photodynamic therapy (PDT), are widely used to treat infections and exhibit a distinct advantage over conventional chemical antimicrobial agents 27. Considering the complex structure of the eye, a bactericidal strategy of mild temperature photothermal assisted therapy, which remains the antibacterial effect but reduces thermal damage to nearby tissues, is more suitable for endophthalmitis 28, 29. PDT, which can produce reactive oxygen species (ROS), also shows antibacterial ability while it can improve the treatment effects and decrease side effects of PTT 30. Consequently, the combination of multiple antibacterial strategies may exhibit better efficiency and potential application prospect.

After cataract surgery, both severe bacterial infection and massive inflammation caused complicated local microenvironment, and are difficult to be cured by the antibiotic treatment. Therefore, effective treatment is imperative to treat and control the endophthalmitis disease 1. Without appropriate diagnosis and treatment, postcataract ocular inflammation can break down the blood-ocular barrier and bring about serious complications like corneal edema, intraocular pressure spikes, cystoid macular edema, posterior capsule opacification and even irreversible vision loss 31, 32. To reduce the damage caused by inflammation, topical corticosteroids and nonsteroidal anti-inflammatory drug are administered by ophthalmologists 33, 34. Bromfenac sodium, as a commonly clinical used nonsteroidal anti-inflammatory drugs with no obvious side effects and exerts superior ability of the treatment for ophthalmic inflammatory disorders and has been used in the clinic for more than 10 years worldwide 35. However, most of the present therapeutic methods mainly focus on the bactericidal effect, so it is urgent to develop a comprehensive treatment for both anti-bacterial and anti-inflammatory effects. Compared with previously reported Ag-based antibacterial core-shell NPs, bromfenac sodium were loaded into novel hollow core-shell nanoparticles could be given the anti-inflammatory ability of NPs 36, 37.

**Scheme 1:**
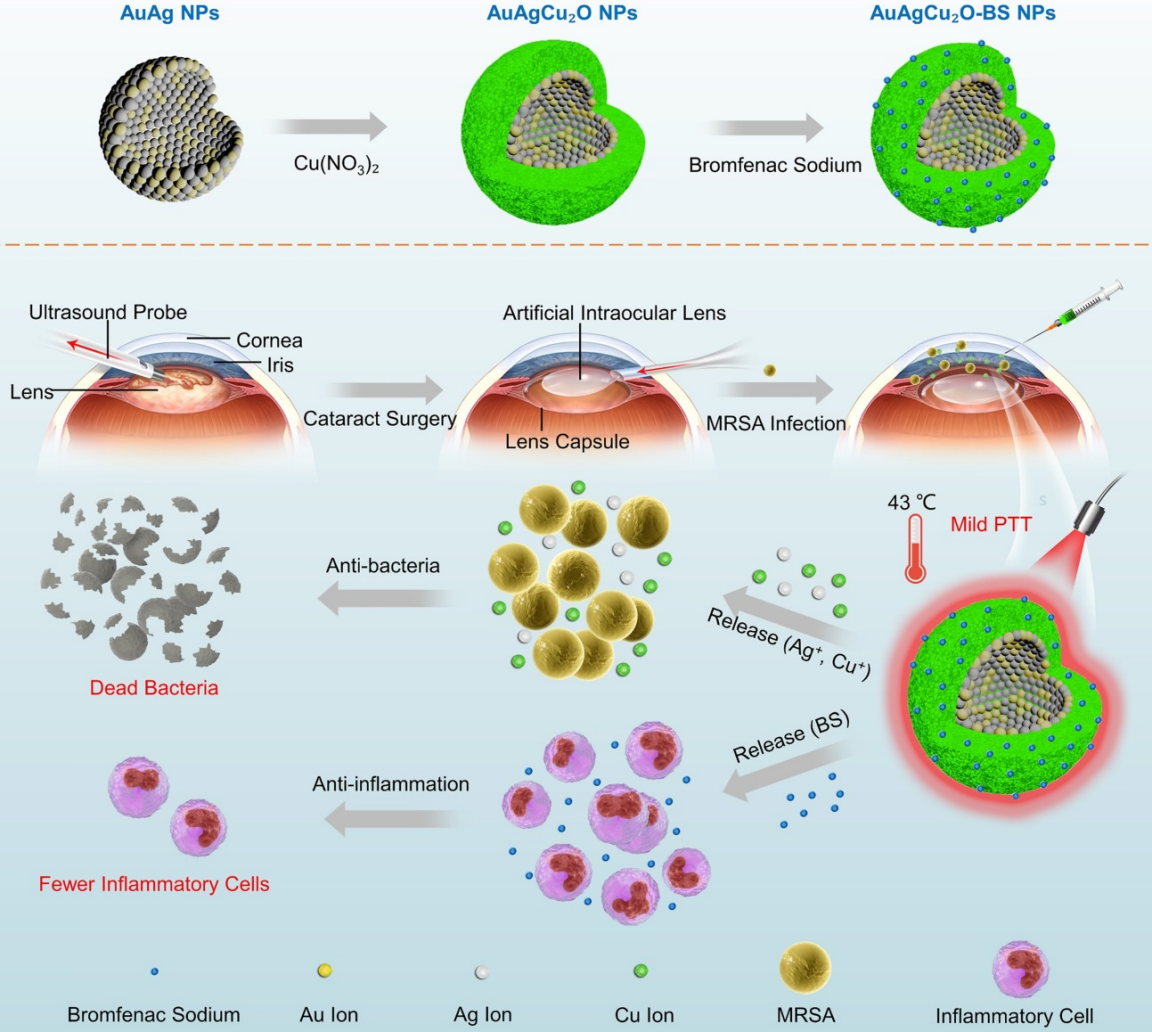
** The schematic illustration of AuAgCu_2_O-BS NPs for the treatment of endophthalmitis after cataract surgery.** On the basis of the hollow AuAg core nanoparticles structure, the nanoparticles were coated with Cu_2_O outer shell layer, and then bromfenac sodium was loaded to develop AuAgCu_2_O-bromfenac sodium core-shell nanosystem. After phacoemulsification, artificial intraocular lens implantation, and the establishment of endophthalmitis, the nanoparticles were injected into the anterior chamber. Upon the near-infrared laser irradiation, AuAgCu_2_O-BS NPs exhibited anti-bacterial and anti-inflammatory effects simultaneously.

In this study, aimed at endophthalmitis after cataract surgery, we developed a novel AuAgCu_2_O-bromfenac sodium nanoparticles (AuAgCu_2_O-BS NPs) which were designed to combine anti-bacterial and anti-inflammatory effects to improve the therapeutic effect of endophthalmitis. As shown in **Scheme 1**, based on the hollow AuAg core nanoparticles structure, the nanoparticles were coated with Cu_2_O outer shell layer, and then bromfenac sodium was loaded to develop AuAgCu_2_O-bromfenac sodium core-shell nanosystem. After phacoemulsification, artificial intraocular lens implantation, and the establishment of endophthalmitis, AuAgCu_2_O-BS NPs were injected into the anterior chamber of rabbits. Assisted with mild photothermal effects, the release of metal ions (Ag^+^ and Cu^+^) could eliminate MRSA and bromfenac sodium could diminish inflammation reaction to achieve anti-bacterial and anti-inflammatory effects simultaneously. Bacterial colony counting, OD_600_ absorption analysis, Live/Dead double staining, ROS staining, transmission electron microscopy, and scanning electron microscopy measurement were used to verify the *in vitro* antibacterial efficacy and possible mechanism of the nanoparticles. Besides, the cell migration test confirmed the promoting healing effect with the mild PTT treatment strategy. As for *in vivo* therapeutic capability, bacterial colony counting, Gram staining, hematoxylin and eosin (H&E) staining, and immunohistochemical staining were applied to demonstrate ideal treatment outcome in a multi-drug resistance (MDR) bacteria-infected postcataract endophthalmitis rabbit model.

## Materials and Methods

The materials and methods used are summarized here. For more details, refer to the Supplementary Methods provided in the SI file.

### Synthesis of AuAgCu_2_O-Bromfenac Sodium Hybrid Core-Shell Nanoparticles

The hollow AuAg nanoparticles were synthesized in advance with the classic method of the Ag nanoparticles templated galvanic replacement reaction and then added into the 10 mL mixed solution of a PVP (M_w_ 40000, 15 mg/mL) and Cu(NO_3_)_2_ (0.1 M, 1 mL) aqueous solution. The solution was stirred for 30 min, and 10 μL of the N_2_H_4_·solution (35 wt%) was added. Then, the yellow-green hollow AuAgCu_2_O nanoparticles were centrifuged and washed. To load the bromfenac sodium, the AuAgCu_2_O dispersion solution was centrifuged to remove the supernatant, and a bromfenac sodium solution (1 mg/mL) was added avoiding light. After stirring for 24 h, the mixed solution was centrifuged, and the free bromfenac sodium in the supernatant was removed. The AuAgCu_2_O-BS NPs were washed thrice and re-dispersed in water for further use.

### Bromfenac Sodium, Au Ion, Ag Ion, and Cu Ion Release Assay

The standard curve of the UV absorbance of different concentrations at 378 nm was drawn by the gradient concentration of bromfenac sodium solution. The AuAgCu_2_O-BS NPs solution was put into a dialysis bag (MWCO = 14 kDa) and dialyzed in DI water. The release of bromfenac sodium from these NPs (1 mg/mL) with/without laser (808 nm, 0.75 W/cm^2^) was measured at certain time points (0-72 h per 12 h) from the release medium. The calibration curve of the released bromfenac sodium concentration was drawn according to the Lambert-Beer law and standard curve.

Similarly, the AuAgCu_2_O-BS NPs solution was put into a dialysis bag (MWCO = 14 kDa) and dialyzed in DI water. These NPs (1 mg/mL) were irradiated with/without laser (808 nm, 0.75 W/cm^2^) and the corresponding release medium were collected at certain time points (0-72 h per 12 h). Then the release medium was measured by ICP-MS (PerkinElmer NexION 300X, USA) to evaluate the amount of the Cu ion, Ag ion and Au ion release from AuAgCu_2_O-BS NPs and diffused into medium at different time.

### Establishment of Endophthalmitis after Cataract Surgery Model

All the experiments on animals were accredited by the Institutional Ethics Committee and followed the requirements for the care and use of laboratory animals of Zhejiang University. The female New Zealand White rabbits used in this study were obtained from the Zhejiang Academy of Medical Science (Hangzhou, China), which were 2000 g and between eight and nine weeks. The bacterial strain of MRSA was used for the rabbit endophthalmitis model. The rabbits were anesthetized with an auricular vein injection of sodium pentobarbital (30 mg/kg) and treated with phacoemulsification surgery on the right eyes. Prior to surgery, the surface of the eye was instilled with a drop of Proparacaine Hydrochloride (s.a. Alcon-Couvreur n.v., Belgium) for topical anesthesia. Then, the lens was divided into smaller pieces and removed through anterior capsule continuous circular capsulorhexis and ultrasonic emulsification through a limbus incision. Afterward, the artificial intraocular lens (IOLs) (6 6 VISION TECH Co., Ltd., FV-60A, China) was implanted into the capsular bag. The corneal incision was closed with 10-0 nylon sutures to prevent leakage. The same surgeon performed all the surgeries and procedures. 24 h after surgery, 50 μL aqueous humor was aspirated from the anterior chamber, and 50 μL MRSA suspension (3.0 × 10^5^ CFU/mL) was injected to establish an experimental model of endophthalmitis after cataract surgery.

### *In Vivo* Treatment Effect Analysis

24 h after the injection of MRSA, the rabbits were divided into four groups (n = 3/group) as follows: untreated, AuAgCu_2_O NPs (21.6 μg/mL, 50 μL), AuAgCu_2_O NPs (21.6 μg/mL, 50 μL) irradiated with an 808 nm laser (0.75 W/cm^2^, 10 min), AuAgCu_2_O-BS NPs (21.6 μg/mL, 50 μL), and AuAgCu_2_O-BS NPs (21.6 μg/mL, 50 μL) irradiated with an 808 nm laser (0.75 W/cm^2^, 10 min). AuAgCu_2_O NPs or AuAgCu_2_O-BS NPs were injected into the anterior chamber. To compare the therapeutic efficacy, ophthalmic clinical observations were performed every three days after the injection of the bacterial suspension. The anterior segment images were recorded with a slit lamp image system (6 6 VISION TECH Co., Ltd., YZ5T, China). The Clinical Grading Scale was applied by three independent, masked observers according to the numeric scale based on criteria proposed by Peyman et al 38 (**Table 1**). On day 12, 50 μL aqueous humor was aspirated and spread on LB plates. The number of CFUs was calculated based on CFU emergence. All groups of rabbits were euthanized on the twelfth day, and infected tissue was excised for pathological analysis. For the histopathological analysis, tissue samples were fixed in 4% paraformaldehyde, dehydrated, embedded in paraffin, and sectioned into four μm slices. These tissue slides were stained with hematoxylin and eosin (H&E) and Gram. High-resolution images of all the histological slices were obtained and analyzed via virtual slide microscopy (Olympus VS120, Japan).

### Measurements of IOP

The intraocular pressure (IOP) of New Zealand Rabbits was noninvasively measured with Tono-Pen XL (Reichert TONO-PEN XL Tonometer, USA) every three days. It was calibrated as instructed in the manufacturer's manual before the first use. Before the measurement, the rabbit's right eye was treated with a drop of Proparacaine Hydrochloride (s.a. Alcon-Couvreur n.v., Belgium). The result of the IOP was the average of three measurements for each eye.

## Results and Discussion

### Preparation and Characterization of AuAgCu_2_O-BS NPs

**Figure 1:**
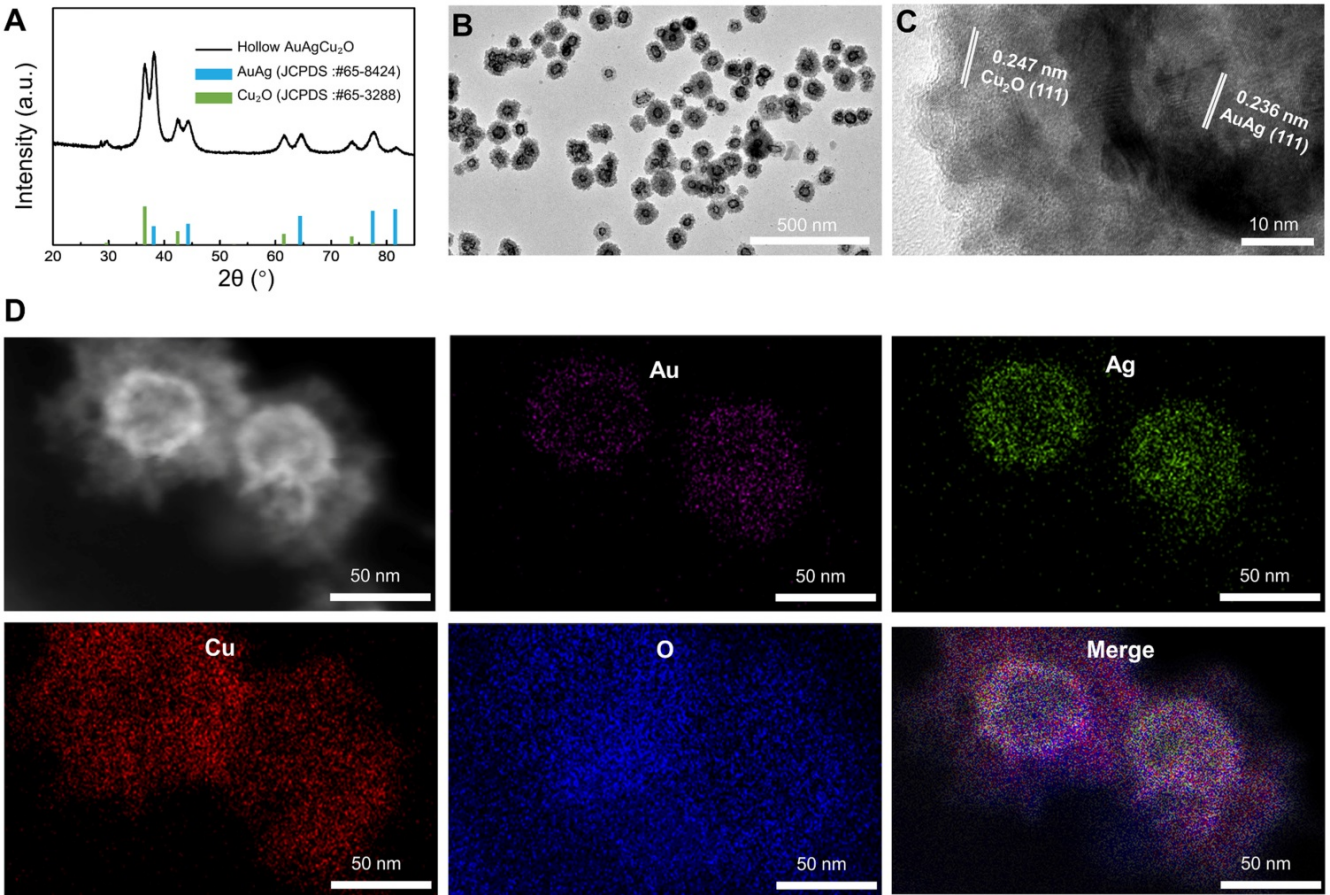
** Morphology and crystal phase characterization of AuAgCu_2_O NPs. (A)** X-ray powder diffraction (XRD) spectrum of AuAgCu_2_O NPs. **(B)** Transmission electron microscopy (TEM) micrographs images of AuAgCu_2_O NPs. **(C)** The HRTEM image of AuAgCu_2_O NPs with core-shell structure in Figure 1A. **(D)** Distribution of Au (purple), Ag (green), Cu (red), and O (blue) elements of AuAgCu_2_O NPs by element mapping.

The AuAgCu_2_O-BS NPs were prepared for endophthalmitis after cataract surgery, which enables the anti-bacterial and anti-inflammatory ability simultaneously. For this purpose, the hollow AuAg nanoparticles were first synthesized by the Ag nanoparticles templated galvanic replacement reaction, and then a Cu_2_O layer was grown on its surface under the support of polyvinylpyrrolidone molecules (PVP). The diffraction peaks in the X-ray powder diffraction (XRD, Figure 1A) verified that these synthetic nanoparticles were composed of AuAg (JCPDS: #65-8424) and Cu_2_O (JCPDS: #65-3288). Furthermore, AuAgCu_2_O NPs with a mean size of 102 nm and a hollow structure were observed by transmission electron microscopy (TEM) (Figure 1B). The high-resolution transmission electron microscopy (HRTEM) image in Figure 1B showed that the lattice fringes presented an AuAg-AuAg spacing of 0.236 nm, which corresponded to the (111) planes of AuAg, while the lattice fringes of Cu_2_O on the interface presented a Cu-Cu spacing of 0.247 nm, which corresponded to the (111) planes of Cu_2_O. TEM elemental mappings (Figure 1D) confirmed again that designed nanosystem was composed of Au, Ag, Cu, and O. Combining above results, the distribution of the elemental showed a core-shell structure that Au and Ag distributed interiorly (hollow AuAg) and Cu and O distributed externally (Cu_2_O).

**Figure 2:**
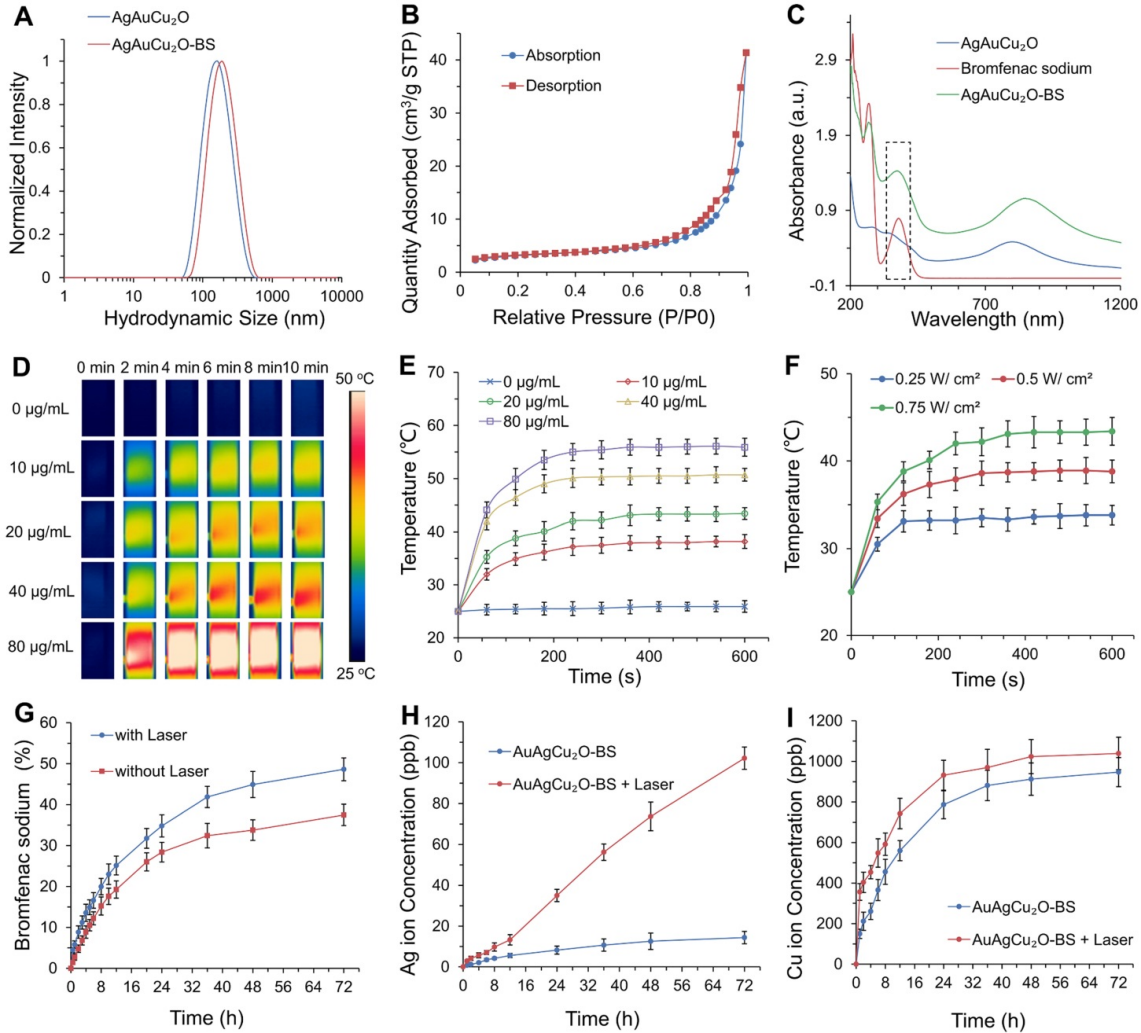
** Drug delivery, release, and photothermal properties of designed hollow AuAgCu_2_O-BS NPs. (A)** The hydrodynamic diameter of AuAgCu_2_O NPs and AuAgCu_2_O-BS NPs measured by dynamic light scattering (DLS). **(B)** BET surface areas of AuAgCu_2_O NPs. **(C)** UV-Vis-NIR absorption spectrum of AuAgCu_2_O-BS NPs. **(D-E)** Thermal images and corresponding temperature change of AuAgCu_2_O-BS NPs with different concentrations under 808 nm NIR irradiation (0.75 W/cm^2^). **(F)** The temperature increment of AuAgCu_2_O-BS NPs with different power densities under the 808 nm laser at the concentration of 20 μg/mL. **(G-I)** Cumulative amounts of bromfenac sodium, Ag ions, and Cu ions released from the AuAgCu_2_O-BS NPs with or without irradiation under the 808 nm laser (0.75 W/cm^2^, 10 min).

Figure 2A showed that the average hydrodynamic size of AuAgCu_2_O NPs was ~164 nm, which was slightly larger than that observed by the TEM due to the low electron density of the extended PVP and their hydration surface. After loading bromfenac sodium, the average hydrodynamic size of AuAgCu_2_O-BS NPs was about 190 nm. The stability was verified in different physiological solutions (Figure S1). Brunauer-Emmett-Teller (BET) surface areas (Figure 2B) and pore volume (Figure S2A) were applied to evaluate the capacity of drug loading. The specific surface area was 10.8009 m²/g, and there were numerous pores with a size of 7 nm and some larger pores, which indicated the capacity of drug loading by physical absorption of the nanoparticles (Figure S3). Similar methods of drug delivery have also been widely reported in typical mesoporous silicon materials and other porous nanoparticles 39, 40. Bromfenac sodium showed a characteristic UV-Vis-NIR absorption peak at 378 nm (Figure S2B), and the absorption was linearly changed with the concentration from 0.2441 μg/mL to 250 μg/mL (Figure S2C). In Figure 2C, AuAgCu_2_O-BS NPs showed a strong characteristic absorption peak of the bromfenac sodium at 378 nm, indicating successful loading of bromfenac sodium (the loading efficiency was calculated to be 3.3%, the process of optimizing the loading of bromfenac sodium was shown in Figure S4**.**), and another broad localized surface plasmon resonance (LSPR) absorption band of hollow AuAg-Cu_2_O from 720 nm to 980 nm, which can induce a NIR-laser-driven photothermal effect. The 808 nm-laser was selected because it matched the LSPR absorption peak of AuAgCu_2_O nanoparticles and penetrated deeper than red light or other visible light with less nonspecific photothermal heating of normal cells. Then, the photothermal ability of the AuAgCu_2_O-BS NPs solution with different concentrations and different laser power densities was evaluated (Figure 2D-F, Figure S2D,E). The temperature elevation of the AuAgCu_2_O-BS NPs solution varied rapidly in the first 5 min and then remained roughly stable from 38.2 °C to 55.9 °C with the concentrations from 10 μg/mL to 80 μg/mL after 0.75 W/cm² 808 nm-laser irradiation, whereas that of the pure water was almost unchanged.

The temperature for the solution at the concentration of 20 μg/mL was also adjusted with an increase from 33.5 °C to 44.2 °C by changing the laser power density from 0.25 W/cm² to 0.75 W/cm^2^. It is noteworthy that the mild photothermal-induced temperature below 45 °C could decrease damage to surrounding tissues significantly, while a high temperature can cause severe inflammation and thermal damage to surrounding tissues in some special organs, such as the eye 30.

The sustained release levels of drug or metallic ions are important to ensure treatment effect, so the deliverance behavior of the bromfenac sodium, Au ion, Ag ion, and Cu ion was then measured under the mild photothermal environment. As shown in Figure 2H-I, the release rate was much faster in the first 24 h than in later. Furthermore, the release amount was clearly improved when treated with NIR laser radiation, demonstrating their possible better laser-induced antibacterial and anti-inflammatory effects. As shown in Figure S5, the cumulative released amounts of Au ions were almost 0 ppb due to its high chemical stability which made it hard to be oxidized to ion and went into solution 41. The situation of release in PBS was also tested and it was similar with in DI water (Figure S6). As reported, the bromfenac sodium had some potential side effects of cornea damage 42 and conjunctiva damage 43, while the sustained release of the drug could reduce the side effects 44.

### *In Vitro* Antibacterial Effect of AuAgCu_2_O-BS NPs

**Figure 3:**
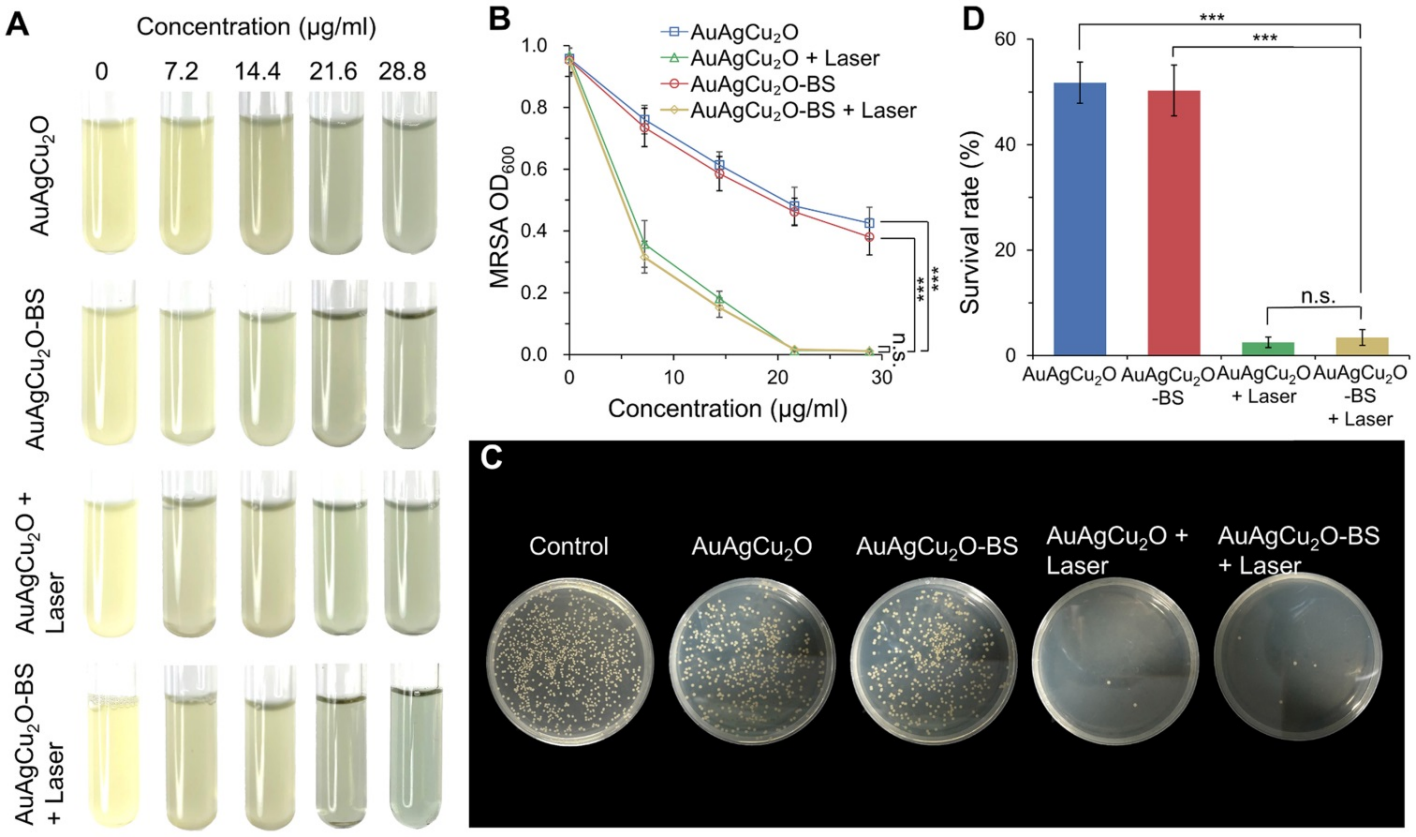
***In vitro* antibacterial effect study. (A)** Images and **(B)** optical density (OD_600_) of MRSA treated with different concentrations of AuAgCu_2_O NPs, AuAgCu_2_O-BS NPs, AuAgCu_2_O NPs with a laser (0.75 W/cm^2^, 10 min), and AuAgCu_2_O-BS NPs with a laser (0.75 W/cm^2^, 10 min). **(C)** Plates images and **(D)** CFU count of MRSA bacterial colonies treated with AuAgCu_2_O NPs, AuAgCu_2_O-BS NPs, AuAgCu_2_O NPs with a laser (0.75 W/cm^2^, 10 min), and AuAgCu_2_O-BS NPs with a laser (0.75 W/cm^2^, 10 min). (***p < 0.001.)

The antibacterial effect of AuAgCu_2_O-BS NPs against methicillin-resistant Staphylococcus aureus (MRSA) was shown in Figure 3A and Figure S7A. The bacteria suspensions' turbidity and optical density value (Figure 3B, Figure S7B) of the AuAgCu_2_O and AuAgCu_2_O-BS NPs without the laser exhibited similar antibacterial ability and was increased when the concentration was increased. However, when the nanoparticles were treated with NIR laser irradiation, the antibacterial ability was enhanced clearly. Almost all the bacteria were killed when the concentration up to 21.6 μg/mL. Plate counting (Figure 3C, and Figure S7C) and survival rates (Figure 3D, and Figure S7D) also showed that AuAgCu_2_O NPs or AuAgCu_2_O-BS NPs with the laser treatment demonstrated more effective function in inhibiting the reproduction of bacteria than that without laser irradiation. In addition, bromfenac sodium, as a type of non-steroid anti-inflammatory drug, did not exhibit an influence on the antibacterial effect of AuAgCu_2_O NPs after the drug loading.

### Possible Antibacterial Mechanism

**Figure 4:**
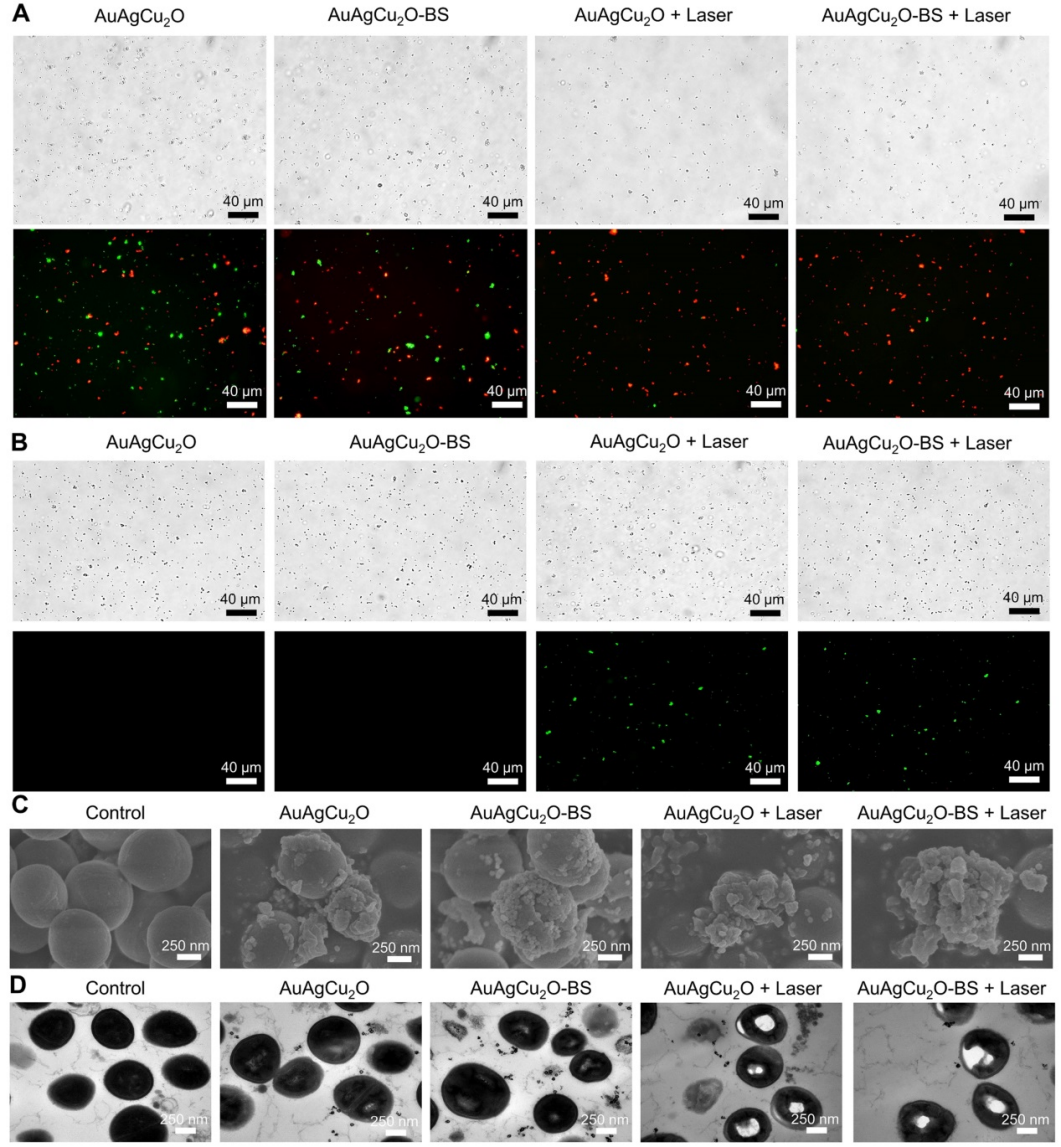
** Live/Dead double staining, ROS, and morphological analysis of antibacterial ability. (A)** Bright-field and fluorescent images of Live/Dead double staining of MRSA treated with AuAgCu_2_O NPs, AuAgCu_2_O-BS NPs, AuAgCu_2_O NPs with a laser (0.75 W/cm^2^, 10 min), and AuAgCu_2_O-BS NPs with a laser (0.75 W/cm^2^, 10 min). Stained by SYTO 9 and PI. **(B)** Bright-field and fluorescent images of ROS levels of MRSA treated with AuAgCu_2_O NPs, AuAgCu_2_O-BS NPs, AuAgCu_2_O NPs with a laser (0.75 W/cm^2^, 10 min), and AuAgCu_2_O-BS NPs with a laser (0.75 W/cm^2^, 10 min). Stained by DCFH-DA. **(C)** SEM micrographs of MRSA treated with AuAgCu_2_O NPs, AuAgCu_2_O-BS NPs, AuAgCu_2_O NPs with a laser (0.75 W/cm^2^, 10 min), and AuAgCu_2_O-BS NPs with a laser (0.75 W/cm^2^, 10 min). **(D)** TEM micrographs of MRSA treated with AuAgCu_2_O NPs, AuAgCu_2_O-BS NPs, AuAgCu_2_O NPs with a laser (0.75 W/cm^2^, 10 min), and AuAgCu_2_O-BS NPs with a laser (0.75 W/cm^2^, 10 min).

The bactericidal function was further verified by a Live/Dead double staining assay. As shown in Figure 4A and Figure S8A, there were a large number of live bacteria (green fluorescence) after treated with AuAgCu_2_O or AuAgCu_2_O-BS NPs. In the AuAgCu_2_O NPs plus laser and AuAgCu_2_O-BS NPs plus laser groups, the percentage of dead bacteria were clearly increased, and almost no bacteria survived. Oxidative stress was recognized as an effective method to kill bacteria 45. The generation of the ROS level was detected by DCFH-DA, which could be oxidized to generate fluorescent products. In Figure 4B and Figure S8B, MRSA treated with AuAgCu_2_O NPs or AuAgCu_2_O-BS NPs could hardly produce the ROS effect. In contrast, NIR laser-irradiated AuAgCu_2_O NPs or AuAgCu_2_O-BS NPs generated apparent ROS signals, which indicated that NIR laser could stimulate the generation of ROS. The morphological characterization and integrity of bacteria were also analyzed through scanning electron microscopy (SEM) and TEM measurements. As shown in Figure 4C and Figure S9, in the SEM images, the bacteria without treatment showed unbroken spherical structures and smooth surfaces of MRSA. AuAgCu_2_O NPs or AuAgCu_2_O-BS NPs could destroy the bacteria, and holes and cracks on the surface could be observed, which could lead to the release of intracellular components. NIR laser-irradiated AuAgCu_2_O NPs or AuAgCu_2_O-BS NPs exhibited much better bactericidal effects in that the bacterial membrane was clearly broken, and the bacterial membrane was completely shrunken. Besides, Energy-dispersive X-ray spectroscopy (EDS) analysis showed that there were nanoparticles on the surface of bacteria. This kind of interaction between NPs and the cell membrane might affect the permeability and integrity of bacteria. As shown in TEM images (Figure 4D), the cytoplasm of the bacteria became nonuniform when treated with AuAgCu_2_O NPs or AuAgCu_2_O-BS NPs, while irregular aggregated districts appeared in the cytoplasm with the laser. EDS analysis showed that there was almost no Au, Ag and Cu in the bacteria without treatment. By contrast, after the bacteria cultured with the AuAgCu_2_O-BS NPs, EDS analysis showed the presence of Au, Ag and Cu in the bacteria (red rectangles), confirming the intrusion of released metal ions (Figure S10). In general, the morphological analysis was consistent with the results mentioned and proved the excellent antibacterial effect of AuAgCu_2_O-BS NPs. In this study, AuAgCu_2_O-BS NPs-mediated phototherapy, including photodynamic therapy and mild temperature photothermal therapy, exhibited a superior bactericidal function. Multiple antibacterial mechanisms of nanomaterials were reported, such as damage to the bacterial membrane, DNA damage, RNA effluxes, oxidative stress, photothermal antibacterial, inhibition of energy metabolism, and so on 25, 46-49. Furthermore, the released Silver ion 50 and Copper ion 51 could also kill bacteria and the antibacterial activity of AuAg NPs was tested (Figure S11). Besides, the release of ions was promoted by irradiation which could enhance the antibacterial effect. In general, the combined effects of multiple mechanisms were the reason for the powerful antibacterial ability.

### *In Vitro* Cell Cytotoxicity, Cell Migration and Anti-inflammatory Effect

**Figure 5:**
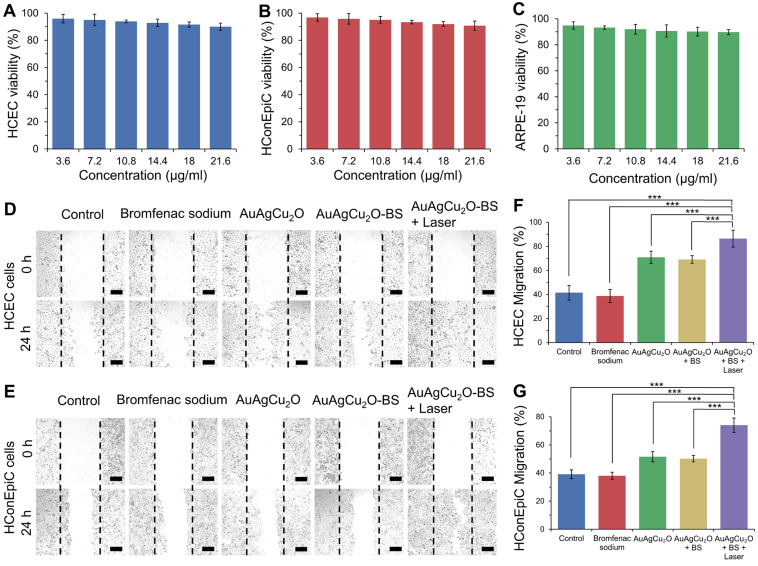
** Cytotoxicity and promoting healing effects study. (A-C)** Cell viability of HCEC, HConEpic, and ARPE-19 incubated with different AuAgCu_2_O-BS NPs concentrations for 48 h with laser irradiation (0.75W/cm^2^, 10 min). **(D,E)** Images of HCEC and HConEpic after treatment with bromfenac sodium, AuAgCu_2_O NPs, AuAgCu_2_O-BS NPs, and AuAgCu_2_O-BS NPs with a laser (0.75W/cm^2^, 10min) for 24h, scale bar=400µm. **(F,G)** Quantification of HCEC and HConEpic cell migration. (***p<0.001.)

*In vitro* cell viability was verified with three types of ophthalmological cells, including human corneal epithelial cells (HCEC), human conjunctival epithelial cells (HConEpic), and retinal pigment epithelia ARPE-19 cells. The cell viability of these three kinds of cells was above 85% when the concentration of AuAgCu_2_O-BS NPs reached 21.6μg/mL, which demonstrated low cell toxicity (Figure 5A-C).

Since the endophthalmitis is commonly caused by surgery and trauma, the healing promotion was also important for the post-surgery recovery. HCEC and HConEpic were chosen for the *in vitro* scratch assay to evaluate their healing ability after the nanoparticles based treatment. In Figure 5D-G and Figure S12, compared with the group without treatment, the migration capability of the cells treated with bromfenac sodium did not exhibit a significant difference for the two types of cells. When treated with nanoparticles (AuAgCu_2_O NPs or AuAgCu_2_O-BS NPs), the cells exhibited better migration capabilities, and migration capabilities of AuAgCu_2_O-BS NPs were further improved when combined with NIR laser irradiation. As we known, the copper ion was demonstrated with the excellent capability to improve wound healing 52. As the important parts of the eye, the wound of tissues such as cornea and iris can easily influence the vision and refraction so the protomting healing effect is also very beneficial for the treatment of the endophthalmitis. As mentioned above, the copper ion could be sustained released from AuAgCu_2_O-BS NPs under the laser irradiation, and then could promote wound healing after the surgery.

*In vitro* anti-inflammatory effect of AuAgCu_2_O-BS NPs was also tested. In Figure S13, the result showed LPS could induce the inflammation of cells and the production of inflammatory cytokines including IL-1β and IL-6 was enhanced. When added with bromfenac sodium, the production of inflammation was significantly decreased which inferred the anti-inflammatory effect of bromfenac sodium. Besides, AuAgCu_2_O NPs did not show an obvious anti-inflammatory effect while the inflammation was not enhanced either. AuAgCu_2_O-BS NPs showed the good anti-inflammatory effect compared with LPS plus PBS group while AuAgCu_2_O-BS NPs with irradiation groups performed better.

### *In Vivo* Treatment Effect of Endophthalmitis after Cataract Surgery Model

**Figure 6:**
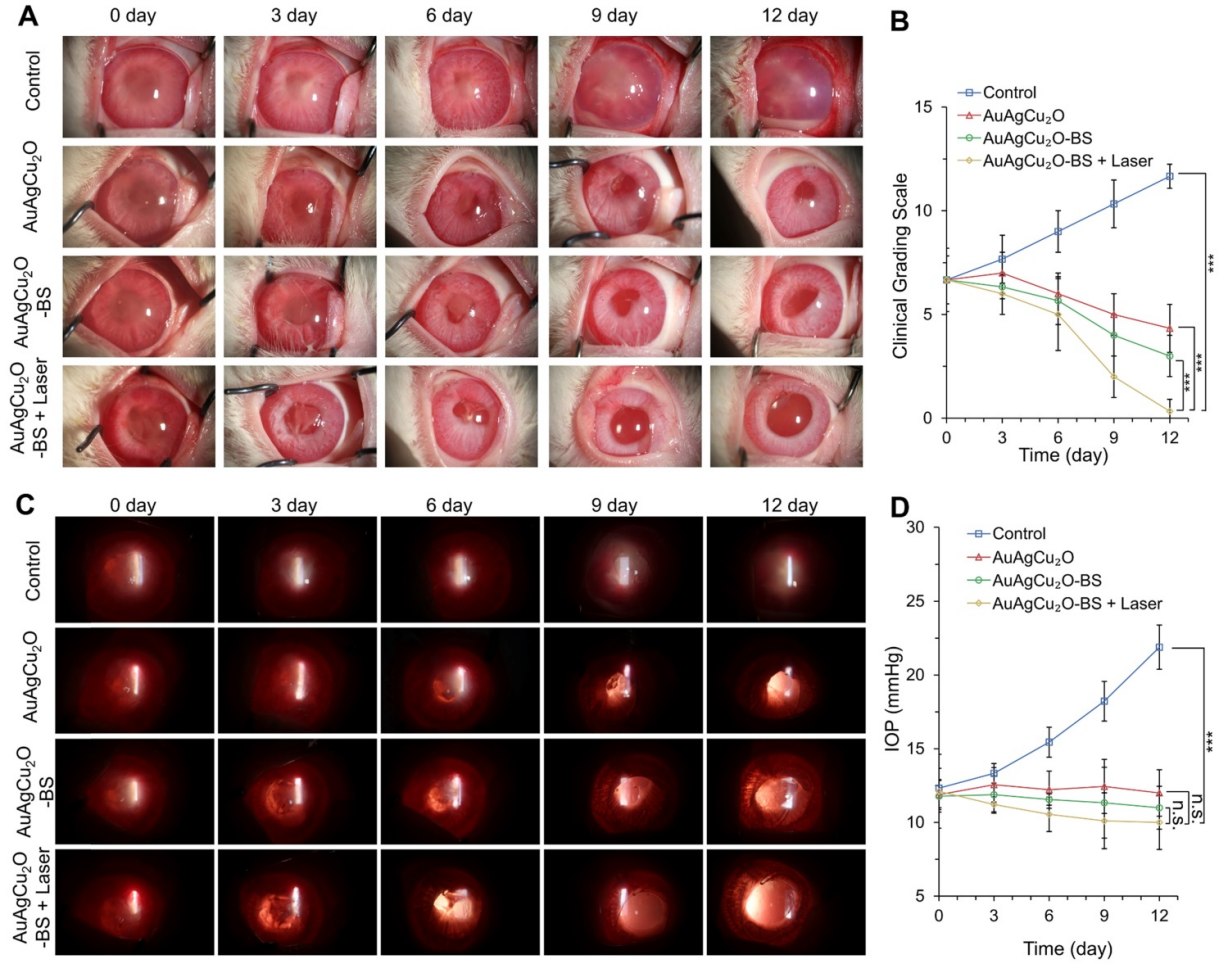
***In vivo* therapeutic effect analysis.** The infected eyes were treated with AuAgCu_2_O NPs, AuAgCu_2_O-BS NPs, and AuAgCu_2_O-BS NPs with a laser (0.75 W/cm^2^, 10 min) in New Zealand rabbits of an MRSA-infected endophthalmitis model after cataract surgery, respectively (0, 3, 6, 9, and 12 days). **(A)** Photographs of slit lamp diffuse illumination. **(B)** The ophthalmological clinical grading scale of endophthalmitis. **(C)** Photographs of slit lamp retroillumination. **(D)** Measurement of intraocular pressure after treatment. (***p < 0.001.)

Since the recovery of the post-cataract surgery endophthalmitis was affected by surgery wounds, bacterial infection, and consequent inflammation simultaneously, the anti-bacterial effect combined anti-inflammatory capability of the treatment should be considered to improve the therapeutic effects. The treatment activity of endophthalmitis after cataract surgery was further evaluated in the MRSA-infected rabbit model. First, the rabbits underwent phacoemulsification and IOL implantation, and then the bacteria suspension was injected into the anterior chamber. Before the treatment, the inflammatory response, including turbid, exudation, and other symptoms, appeared similarly in all groups, which indicated that endophthalmitis occurred. As shown in Figure 6A and Figure S14A, a slit lamp diffuse illumination of the anterior segment images was recorded during the treatment. The inflammation of the control group was gradually aggravated, and fibrin exudation accumulated in the anterior chamber. Neovascularization, opacification of the cornea, conjunctival and congestion hyperemia, and edema of the iris were observed. On day 12, hypopyon clearly appeared, and purulent secretion increased (clinical grading scale: 11.667 ± 0.577). The group treated with AuAgCu_2_O NPs without laser irradiation, AuAgCu_2_O NPs with laser irradiation and AuAgCu_2_O-BS NPs without laser irradiation exhibited moderate therapeutic effects (clinical grading scale: AuAgCu_2_O NPs, 4.333 ± 1.154; AuAgCu_2_O NPs with laser irradiation, 3.333 ± 0.578; AuAgCu_2_O-BS NPs 2.667 ± 1.000), and the condition of the endophthalmitis did not worsen. The AuAgCu_2_O-BS NPs treatment group showed better inflammatory response with less fibrin exudation, less edema, and more regular pupils. However, the group treated with AuAgCu_2_O-BS NPs upon NIR laser-irradiation achieved the best therapeutic effect and almost completely recovered (clinical grading scale: 0.333 ± 0.577). On day 12, there was no symptom of inflammation or infection, and the IOL could be observed clearly, which meant the eye reverted to transparency. The ophthalmological clinical grading scale also indicated that the inflammation was reduced effectively after the AuAgCu_2_O-BS NPs upon NIR laser-irradiation treatment (Figure 6B, Figure S14B). In Figure 6C, images of slit lamp retroillumination, which indicated an intraocular situation and transparency by the reflection on the retina, were also recorded to evaluate the therapeutic effect. Compared with other groups, the group treated with AuAgCu_2_O-BS NPs plus NIR laser-irradiation reached an optimal effect, including less exudation and sooner transparency. As a kind of bacteria infection, antibacterial treatment was most important for endophthalmitis. In the early stage of treatment, the photothermal effect of irradiation which could lead to the topical recruitment of inflammatory cells might enhance the antibacterial effect 53. However, the inflammatory response caused by bacteria in the eye could continue for five to seven days after the bacteria have been killed by antibiotics which would seriously influence prognosis 54. In the later stage, on the base of the excellent antibacterial effect, inflammation reaction was gradually diminished with the decline of photothermal effect and sustained release of anti-inflammatory drug. Therefore, the combined anti-bacterial anti-inflammatory capability of the AuAgCu_2_O-BS NPs could effectively eradicate the bacteria, control inflammation and prevent tissue damage to synthetically treat the endophthalmitis. In addition, the IOP was measured every three days, and the IOP of the group without treatment gradually increased (Figure 6D, Figure S14C); however, it remained stable when treated by AuAgCu_2_O NPs, AuAgCu_2_O NPs with laser irradiation or AuAgCu_2_O-BS NPs and slightly decreased with AuAgCu_2_O-BS NPs upon NIR laser-irradiation during the 12-day treatment, which indicated that the treatment of nanoparticles would not lead to the obstruction of the aqueous inflow.

### Etiological and Inflammatory Pathologic Analysis

**Figure 7:**
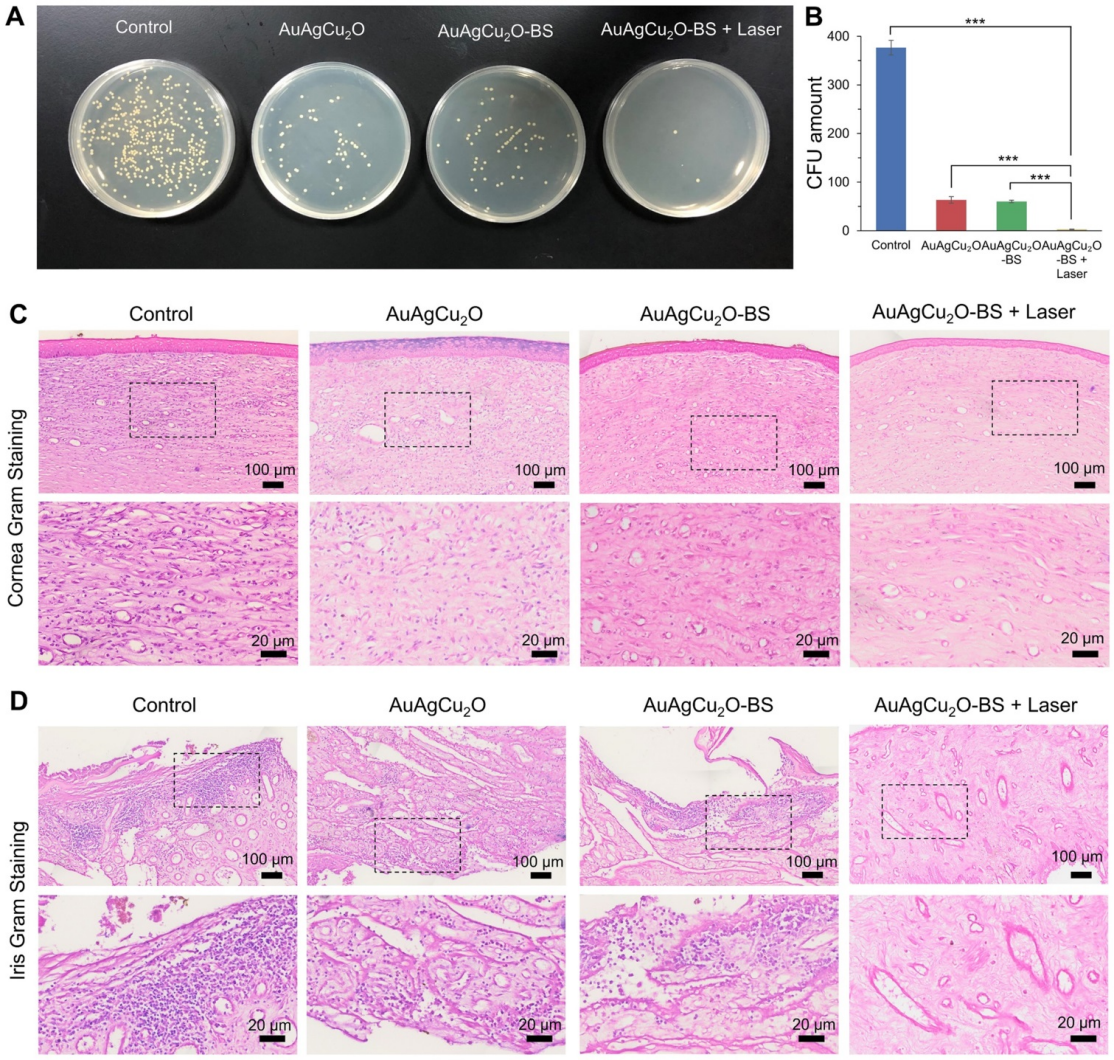
** Etiological analysis of targeting effect at day 12 after treatment. (A)** Photographic images of bacterial colonies and **(B)** counting numbers in aqueous humor after treatment. Gram staining analysis of MRSA in the cornea **(C)** and iris **(D)** indicated the number of Gram-positive cocci decreased. The parts framed with the black dotted line were enlarged, respectively. (***p < 0.001.)

On day 12, the aqueous humor was aspirated for the bacteria culture on LB plates. The group treated with AuAgCu_2_O-BS NPs with laser treatment exhibited excellent bactericidal ability. The combined treatment demonstrated significant antibacterial effects (p < 0.001 vs all other 3 groups), which was consistent with the result *in vitro* (Figure 7A-B, Figure S14D-E). AuAgCu_2_O without NIR irradiation could also release Ag ion and Cu ion and the released ion exhibited antibacterial activity which showed significant difference compared with the control group 20. The therapeutic effects were further verified with immunohistological analysis of the cornea and iris tissue slices. In Figure 7C,D and Figure S14F, a large number of Gram-positive cocci were found by Gram staining of the cornea and iris tissue for the control group. The cocci could be reduced by AuAgCu_2_O NPs, AuAgCu_2_O NPs with laser irradiation or AuAgCu_2_O-BS NPs treatments. However, almost no bacteria could be observed in the group treated with AuAgCu_2_O-BS NPs upon NIR laser irradiation.

**Figure 8:**
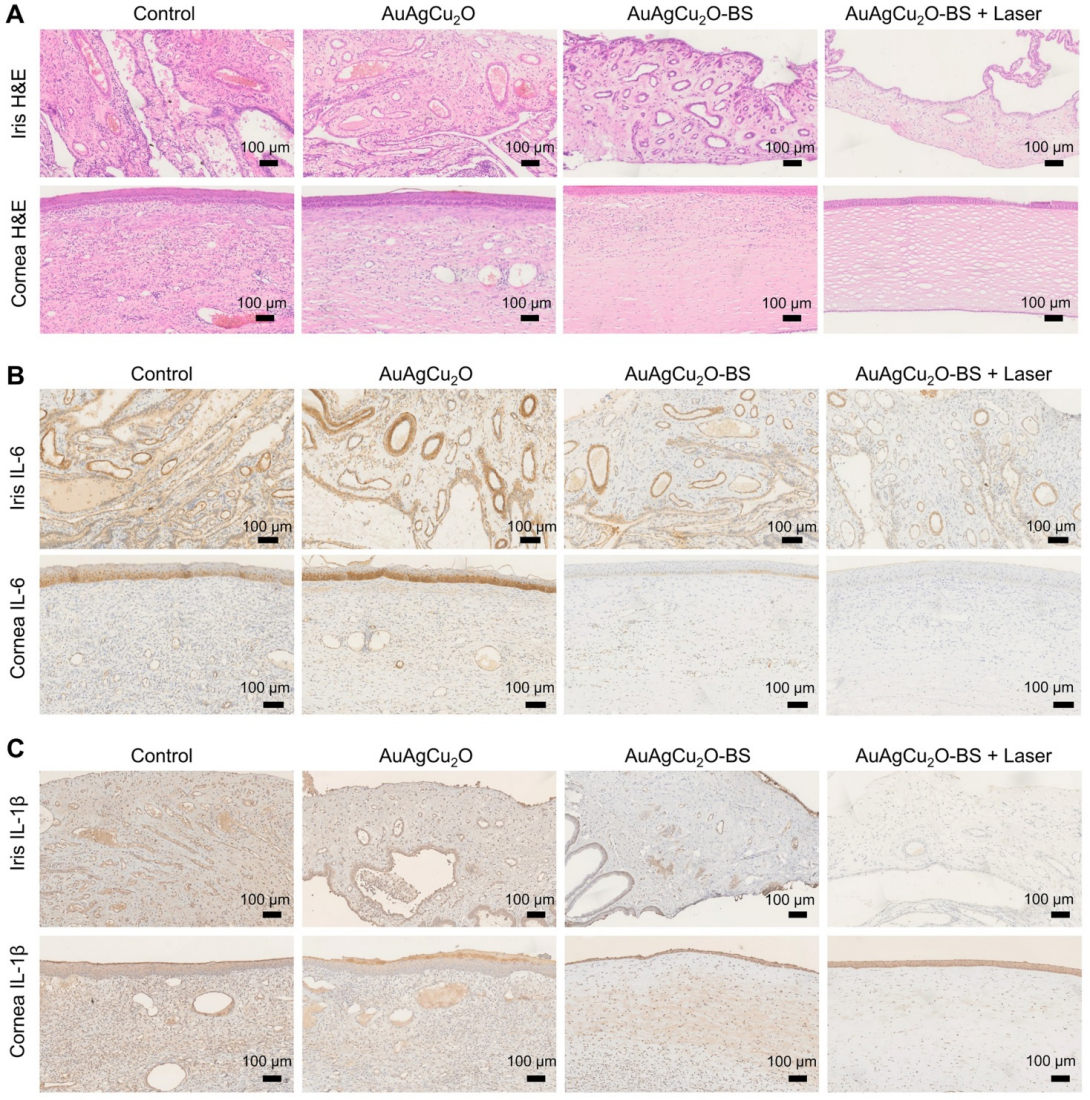
** Pathologic histology analysis. (A)** H&E staining analysis of the cornea and iris indicated the inflammation reaction was inhibited with the AuAgCu_2_O-BS plus laser treatment. Immunohistochemical staining analysis of IL-6 and IL-1β **(C)** in the cornea and iris indicated the obviously decrease of the cytokine after the AuAgCu_2_O-BS + Laser treatment.

For the H&E staining result (Figure 8A, Figure S14G and Figure S15A-B), severe infiltration of several inflammatory cells and an irregular structure were observed in the control group. Almost no inflammatory cells were found in the group with AuAgCu_2_O-BS NPs upon NIR laser-irradiation, and no structural abnormalities were observed, indicating no severe inflammation and damage under the mild photo-induced temperature during the treatment. On the other hand, immunohistochemical staining of cytokine, including IL-1β and IL-6, whose expression increased during inflammation 55, also validated the therapeutic effect (Figure 8B-C, Figure S7G and Figure S15C-D). The expression of proinflammatory cytokines IL-1β and IL-6 was significantly upregulated in endophthalmitis and was relevant to the severity 56. Compared with the control group, the expression of inflammatory factors including IL-1β and IL-6 decreased in the group treated with AuAgCu_2_O NPs, while the expression was lower when treated with AuAgCu_2_O NPs with laser irradiation and AuAgCu_2_O-BS NPs. For the group treated with AuAgCu_2_O-BS NPs upon NIR laser irradiation, the expression of the IL-1β and IL-6 was lowest, which indicated the best therapeutic effect and the least inflammatory reaction. In general, the anti-bacterial and anti-inflammatory ability of AuAgCu_2_O-BS NPs was improved when treated with NIR laser irradiation. In addition, the application prospect of phototherapy demonstrates some advantages for the treatment of ophthalmic diseases. Therefore, AuAgCu_2_O-BS NPs applied for endophthalmitis after cataract surgery exhibited a superior effect and broad prospects.

### Biosafety Study of AuAgCu_2_O-BS NPs

**Figure 9:**
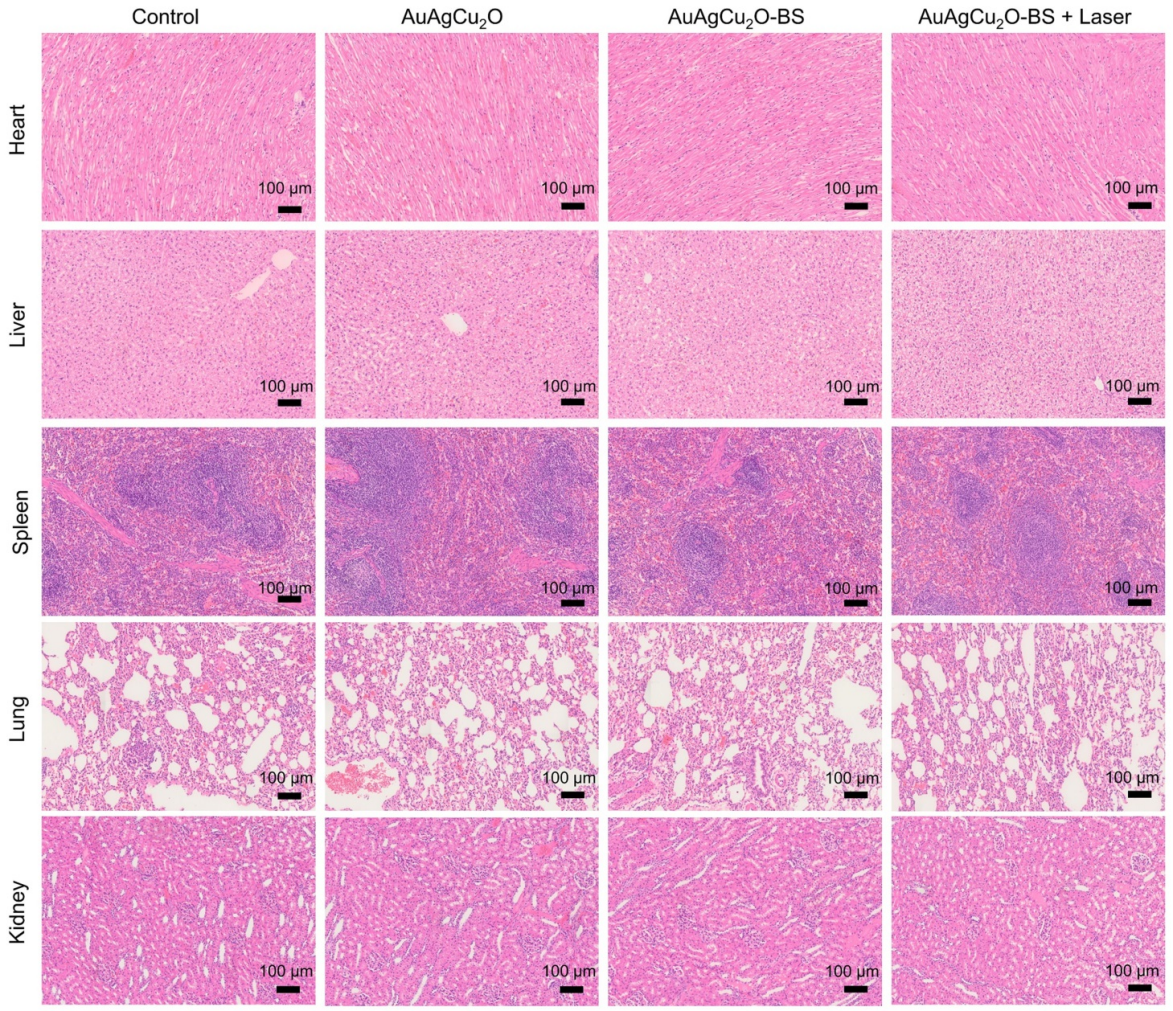
** Preliminary main visceral organs toxicity study.** Toxicological analysis of H&E staining of main visceral organs (heart, liver, spleen, lungs, and kidneys).

**Figure 10:**
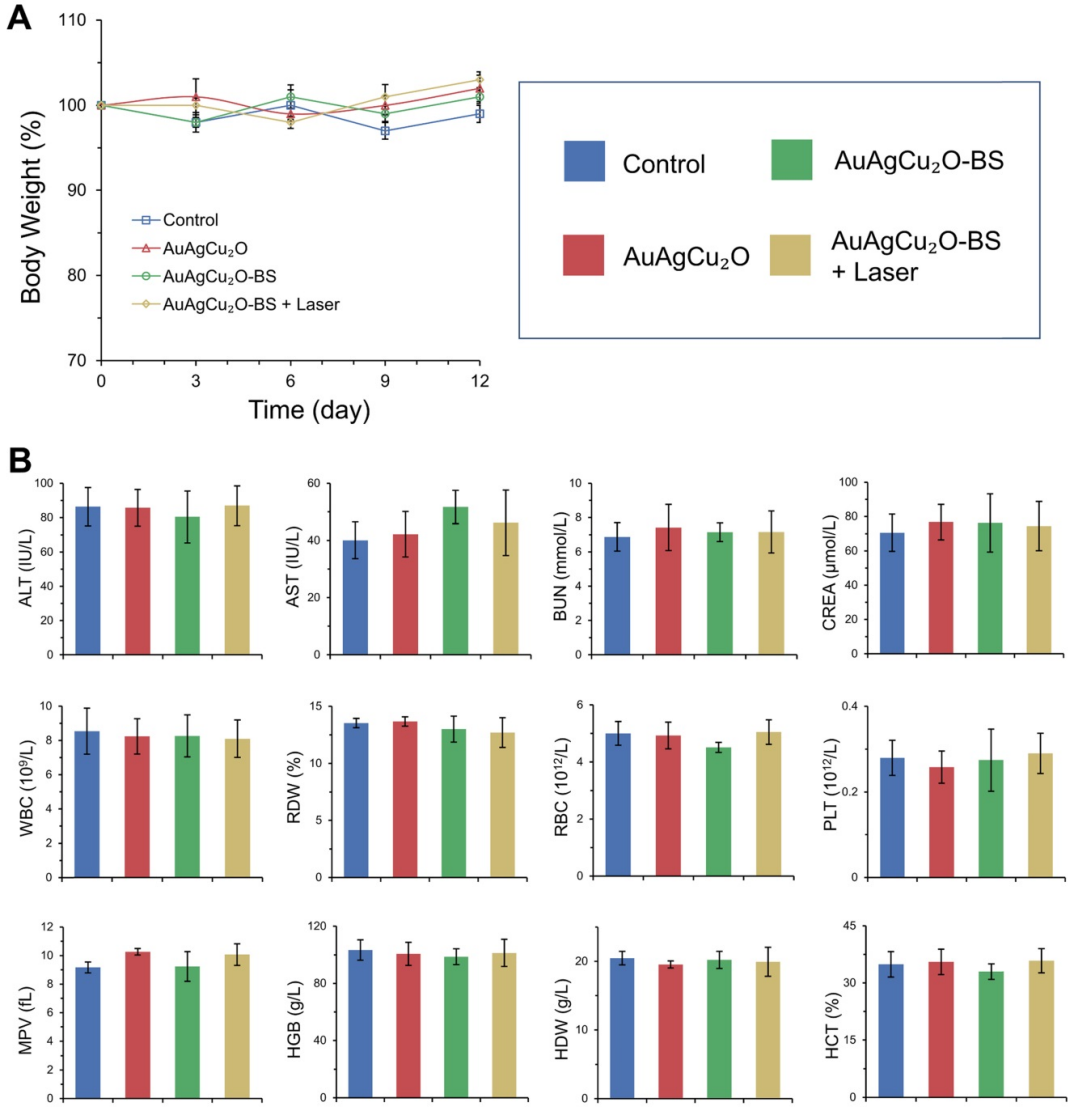
** Changes of body weight (A) with different treatments and blood biochemistry and blood routine examination analyses (B)**. ALT (alanine transferase), AST (aspartate transferase), BUN (blood urea nitrogen), CREA (creatinine), WBC (white blood cells), RDW (red cell distribution width), RBC (red blood cells), PLT (blood platelet), MPV (mean platelet volume), HGB (hemoglobin), HDW (hemoglobin distribution width), and HCT (hematocrit).

Preliminary toxicity of the AuAgCu_2_O-BS NPs was investigated to evaluate their biosafety by H&E staining analysis of major organs, including heart, liver, spleen, lung, and kidney. As shown in Figure 9 and Figure S16A, the H&E staining result showed that the AuAgCu_2_O-BS NPs with laser treatment did not exhibit significant histological differences. The body weight of the rabbits remained stable during treatments and was not recognized to be different between the four groups (Figure 10A, Figure S16B). Moreover, the routine blood examination and the liver and kidney function evaluations were in the normal range (Figure 10B) and there was no death during the treatments. As shown in Figure S17, the NPs did not damage red blood cells (percent hemolysis % <5%) indicating the reliable blood biocompatibility 57. The metabolism of AuAgCu_2_O-BS NPs in rabbits was measured by ICP-MS. As shown in Figure S18, after 6 days, the concentrations of NPs in the eye, liver and feces were higher than other tissues and after 12 days the total concentrations were lower. After a month almost all nanoparticles were metabolized. The results indicated the NPs could be removed from eyes and mainly metabolized by the liver while the H&E staining of the eye and liver did not show obvious change which also indicated the biosafety. Overall, all the results mentioned demonstrated that AuAgCu_2_O-BS NPs applied for endophthalmitis are safe and superior in biosafety, which has laid the foundation for clinical application.

## Conclusion

In summary, we developed an AuAgCu_2_O-BS nanosystem to eliminate MDR bacteria, alleviate inflammation, and protect the ocular thermal damage for postcataract endophthalmitis. We found that the released metal ions from the AuAgCu_2_O-BS NPs could kill the clinical MDR bacteria (MRSA) effectively both *in vitro* and *in vivo*. Meanwhile, the delivered bromfenac sodium to the disease sites from the nanosystem ensured the anti-inflammatory effect and alleviate the relative symptoms to improve the prognosis. Importantly, mild temperature photothermal treatment is able to control the thermal damage to the surrounding ocular structure. Moreover, AuAgCu_2_O-BS NPs did not influence intraocular pressure and showed no significant toxicity after the treatment. Thus, we have demonstrated a promising nanosystem to provide the antibacterial and anti-inflammatory effects to treat postcataract endophthalmitis, and this strategy may contribute to an alternative treatment in the future clinical application.

## Supplementary Material

Supplementary methods and figures.

## Figures and Tables

**Table 1:** Clinical Grading Scale

Score	Conjunctiva	Cornea	Iris	Vitreous Body
**0**	Normal	Clear	Normal	Clear
**1**	Mild edema	Focal edema	Mild hyperemia	Areas of vitreous haze, some fundus details visible, good red reflex
**2**	Edema, mild hyperemia, slight exudate	Diffuse edema	Marked hyperemia	Moderate Vitreous haze, fundus details not clear, partial red reflex
**3**	Edema, mild hyperemia, heavy exudate	Opaque	Marked hyperemia, engorged vessels, synechia, irregular pupil	No red reflex

## Abbreviations

AuAgCu_2_O-BS NPs: AuAgCu_2_O-bromfenac sodium nanoparticles
BS: bromfenac sodium
MRSA: methicillin-resistant *Staphylococcus aureus*
PTT: photothermal therapy
PDT: photodynamic therapy
ROS: reactive oxygen species
OD: optical density
MDR: multi-drug resistance
PVP: polyvinyl pyrrolidone
DI water: deionized water
IOLs: intraocular lens
H&E: hematoxylin and eosin
CFU: colony-forming units
IOP: intraocular pressure
XRD: X-ray powder diffraction
SEM: scanning electron microscopy
TEM: transmission electron microscopy
HRTEM: high-resolution transmission electron microscopy
BET: Brunauer-Emmett-Teller
LSPR: localized surface plasmon resonance
UV: ultraviolet
NIR: near-infrared ray
DCFH-DA: 2,7-dichlorodi-hydrofluorescein diacetate
HCEC: human corneal epithelial cells
HConEpic: human conjunctival epithelial cells
IL: Interleukin
