# Supplementary material for: Mild temperature photothermal assisted anti-bacterial and anti-inflammatory nanosystem for synergistic treatment of post-cataract surgery endophthalmitis
Source: Theranostics. 2020 Jul 9;10(19):8541–57. doi: 10.7150/thno.46895 (PMC7392004; doi:10.7150/thno.46895)
Supplement: Supplementary file 1 — Supplementary methods and figures. [file thnov10p8541s1.pdf]

Supporting Information

Mild temperature photothermal assisted anti-bacterial and anti-inflammatory nanosystem for synergistic treatment of post-cataract surgery endophthalmitis

Yang Ye[1,3,*], Jian He[1,2,*], Yue Qiao[1,2,*], Yuchen Qi[1,2,*], Hongbo Zhang[4,*], Hélder A. Santos[5,9,*], Danni Zhong[1,2], Wanlin Li[1,2], Shiyuan Hua[1,2], Wei Wang[1,3], Andrzej Grzybowski[8], Ke Yao[1,3,#], and Min Zhou [1,2,6,7,#]

[1] Eye Center, The Second Affiliated Hospital, Zhejiang University School of Medicine, Hangzhou 310009, China.

[2] Institute of Translational Medicine, Zhejiang University, Hangzhou, 310009, China.

[3] Zhejiang Provincial Key Lab of Ophthalmology, Hangzhou, 310009, China.

[4] Department of Pharmaceutical Science, Åbo Akademi University; Turku Bioscience Center, University of Turku and Åbo Akademi University, FI-20520, Finland.

[5] Drug Research Program, Division of Pharmaceutical Chemistry and Technology, Faculty of Pharmacy, University of Helsinki, FI-00014, Finland.

[6] Key Laboratory of Cancer Prevention and Intervention, National Ministry of Education, Zhejiang University, Hangzhou, 310009, China.

[7] State Key Laboratory of Modern Optical Instrumentations, Zhejiang University, Hangzhou, 310058, China.

[8] Department of Ophthalmology, University of Warmia and Mazury, Olsztyn, Poland.

[9] Helsinki Institute of Life Science (HiLIFE), University of Helsinki, FI-00014 Helsinki, Finland.

[*] These authors contributed equally to this work.

[#] **Address correspondence to**

zhoum@zju.edu.cn (Min Zhou);

xlren@zju.edu.cn (Ke Yao)

Materials.

All chemicals except bromfenac sodium were purchased from Sigma-Aldrich Corporation and used without further purification. Bromfenac sodium was obtained from Senju Pharmaceutical Co., Ltd. Methicillin-resistant Staphylococcus aureus (MRSA, ATCC 43300), human corneal epithelial cells (HCEC, ATCC PCS-700-010), and human retinal pigment epithelial cells (ARPE-19, ATCC CRL-2302) were obtained from American Type Culture Collection (VA, USA). Human conjunctival epithelial cells (HConEpic, Zhen Shanghai, and Shanghai Industrial Co., Ltd. HZC621) were obtained from Zhen Shanghai and Shanghai Industrial Company (Shanghai, China). New Zealand White rabbits used in this study were obtained from the Zhejiang Academy of Medical Science (Hangzhou, China).

Experiment Methods.

Characterization.

The morphologies of $AuAgCu_2O$ NPs were measured using Tecnai 20 transmission electron microscopy (TEM, FEI Tecnai F20, USA). The absorption of $AuAgCu_2O$-BS NPs were recorded using a UV−vis spectrometer (SHIMADZU UV-2600, Japan). Hydrodynamic size measurements were performed at room temperature by a dynamic light scattering system (Malvern Panalytical Zetasizer Nano ZS90, UK). XRD patterns were characterized by an x-ray diffractometer (Panalytical X'PERT PRO, Netherl1.0ands). The mass ratios of the metal components (Au, Ag, and Cu) in the nanoparticles evaluated by ICP-MS were 18.3:12.3:69.4.

Photothermal Conversion Effect.

The photothermal effect of NPs induced by the 808-laser system (Changchun Optoelectronics MDL-N-808-10W, China) in vitro or in vivo was examined. The experimental conditions were performed at various power densities (0.25, 0.50, and 0.75 W/cm^2) and $AuAgCu_2O$-BS NPs concentrations (0, 10, 20, 40, and 80 μg/mL) with a duration of 10 min. The infrared thermal imaging camera (FLIR A350, USA) recorded corresponding temperature changes.

In Vitro Antibacterial Activity Analysis.

The HCEC were cultured in DMEM/F12 (Gibco, CA, USA) with 15% fetal bovine serum (FBS) (Gibco, CA, USA). The ARPE-19 were cultured in DMEM/F12 with 10% fetal bovine serum (FBS). The HConEpic were cultured in RPMI-1640 medium (Gibco, CA, USA) with 10% fetal bovine serum (FBS). All the cells were passaged with 0.25% trypsin and 0.02% ethylene diamine tetra-acetic acid (EDTA) (Gibco, CA, USA) every three days. MRSA was selected to verify the antibacterial effects of $AuAgCu_2O$ Nanoparticles (NPs) in addition to Near-Infrared Ray (NIR) laser irradiation. Upon laser irradiation (808 nm, 0.75 W/cm^2, 10 min), bacteria were cultured at 37 °C for 24 h in a lysogeny broth (LB) medium with a series of concentrations of $AuAgCu_2O$ NPs (0, 7.2, 14.4, 21.6, and 28.8 μg/mL). Then, the upper suspension was added into a 96-well plate, and the 600 nm wavelength optical density value (OD600) was measured by a microplate reader (SpectraMax M5). After being diluted 10^6-fold, the 100 μL suspension was cultured on LB agar plates with a spreader for 24 h at 37 °C. The amount of the colony-forming unit (CFU) was calculated based on CFUs emergence. The laser (0.75 W/cm^2, 10 min), $AuAgCu_2O$ NPs group, $AuAgCu_2O$ NPs plus laser treatment group, $AuAgCu_2O$-BS NPs group, and the blank control group were also examined in the above manner.

Morphological Characterization of Bacteria.

The bacterial suspensions of MRSA (10^9) that were treated with $AuAgCu_2O$-BS NPs (21.6 μg/mL, 0.75 W/cm^2 for 10 min laser irradiation) were collected, centrifuged (3000 rpm, 5 min), and washed with a phosphate buffer (0.1 M, pH = 7.0). The samples were fixed by a 2.5% glutaraldehyde solution in a phosphate buffer (0.1 M, pH 7.0) overnight and washed three times with a phosphate buffer (0.1 M, pH = 7.0). Then, the samples were fixed again with 1% OsO_4 for 1 h and washed with a phosphate buffer (0.1 M, pH = 7.0) three times, followed by dehydrating with a graded series of ethanol (30%, 50%, 70%, 90%, 95%, and 100%) for 15 min. The sample was then dehydrated again in a Hitachi Model HCP-2 critical point dryer. The samples were collected and coated with gold-palladium for the SEM morphological analysis. For the TEM analysis, the process of dehydration part until a graded series of ethanol dehydrated the samples was the same as the procedure for SEM and were transferred to absolute acetone for 20 min. After being placed in a 1:1 mixture of absolute

acetone and the final Spurr resin mixture for 1 h at room temperature, the samples were transferred to a 1:3 mixture of absolute acetone and the final resin mixture for 3 h and then to a final Spurr resin mixture overnight. The samples were stained by uranyl acetate and alkaline lead citrate for 10 min, respectively, and observed for the TEM morphological analysis.

Bacterial Fluorescent Assay.

To further evaluate the bacterial viability and membrane integrity, the bacterial suspension was centrifuged and washed by a 1 mL PBS solution after being treated with $AuAgCu_2O$-BS NPs (21.6 μg/mL, 0.75 W/cm^2 for 10 min laser irradiation). Then, 2 μL of SYTO 9 and propidium iodide (PI) was added and incubated for 20 min at 37 °C in the dark. Fluorescent images were recorded using a fluorescence inversion microscope (Olympus IX71, Japan).

ROS Effect Assay.

The intracellular ROS level was evaluated by the oxidative conversion of cell-permeable 20,70-dichlorodihydrofluorescein diacetate (DCFH-DA). The bacterial suspension was centrifuged and washed by a 1 mL PBS solution after being treated with $AuAgCu_2O$-BS NPs (21.6 μg/mL, 0.75 W/cm^2 for 10 min laser irradiation). Then, the suspension was incubated in a 96-well plate with 1 mL DCFH-DA (10 μM) at 37 °C in 5% CO_2 for 30 min and washed three times with PBS. The fluorescence images were then taken using a fluorescence inversion microscope (Olympus IX71, Japan).

In Vitro Cell Migration.

HCEC and HConEpic were seeded in a 24-well plate (5.0 × 10^4 cells/well) and cultured with an FBS-free medium for 24 h. Each well was scratched with a straight line by a 200 μL pipette tip and washed with PBS to remove debris. Cells were divided into five groups as follows: untreated, Laser (0.75 W/cm^2, 10 min), bromfenac sodium (1.0 mg/mL), $AuAgCu_2O$ NPs (21.6 μg/mL), $AuAgCu_2O$-BS NPs (21.6 μg/mL), and $AuAgCu_2O$-BS NPs (21.6 μg/mL) irradiated with an 808 nm laser (0.75

W/cm^2, 10 min) and incubated at 37 °C with the medium containing 1% FBS for 24 h. Afterward, each well of the cells was photographed, and the migration rate was calculated.

Cytotoxicity Tests.

HCEC, HConEpic, and ARPE-19 were plated in 96-well plates at an initial density of 5 × 10^3 cells/well with a series of concentrations of $AuAgCu_2O$-BS NPs (21.6 μg/mL) upon laser irradiation (808-nm, 0.75 W/cm^2, 10 min). After incubation for 48 h, 10 μL of a 3-[4,5-Dimethylthiazol-2-yl]-2,5-diphenyltetrazolium bromide (MTT, 10 mg/mL) solution was added to each well. After incubation for another 4 h, the medium was replaced by 100 μL of dimethyl sulfoxide (DMSO) to dissolve crystals. Cell viability was measured as a percentage of the absorbance at 570 nm to that of the blank control group without treatment.

In vitro anti-inflammatory Test.

To evaluate the anti-inflammatory capacity of bromfenac sodium in vitro, inflammation of HCEC, HConEpic, and ARPE-19 cells were activated by LPS. Cells were seeded into 96-well plates and were allowed to adhere overnight, followed by treated with LPS + PBS, LPS + bromfenac sodium, LPS + $AuAgCu_2O$ NPs, LPS + $AuAgCu_2O$-BS NPs and LPS + $AuAgCu_2O$-BS NPs +Laser (0.75 W/cm^2, 10 min) for 24 h. Cells without any treatment were used as control. The concentrations of IL-1β and IL-6 in the culturing medium were detected by Elisa kits (USCN Business Co., Ltd., China) according to the manufacturer's instructions and the experiments were carried out in parallel and in triplicate.

Immunohistochemical Analysis.

The slides were obtained as mentioned and incubated in 10% goat serum for 30 min to block non-specific binding sites. After incubation with IL-1β (Abcam, ab8320, 1:100) and IL-6 (Abcam, ab9324,1:250) at 4 °C overnight, the signal-labeled antibody was added to the slides. The slides were stained by diaminobenzidine (DAB) and counterstained with hematoxylin.

Biosafety Study of $AuAgCu_2O$-BS NPs.

In vivo toxicity assays were conducted with New Zealand White rabbits. The blood was drawn for a routine blood examination and liver and kidney functions. Five types of organs, including the heart, lungs, liver, spleen, and kidneys, were excised for pathological analysis. Tissue samples were fixed in 4% paraformaldehyde, dehydrated, embedded in paraffin, and sectioned into 5 μm slices. These slides were stained with hematoxylin and eosin (H&E) for the toxicology analysis.

Hemolytic Test

5 mL fresh blood was collected in anticoagulant tubes and centrifuged at 1500 rpm for 15 min to obtain red blood cells (RBCs). After washed several times with PBS, RBCs were diluted into 50 mL PBS as stock dispersion. 0.2 mL diluted stock suspension was mixed with $AuAgCu_2O$ NPs (7.2, 14.4, 21.6, 28.8 μg/mL) dispersions (0.8 mL in PBS) while DI water and PBS were served as the positive (+) and negative controls (-) group, respectively. After incubated at room temperature for 4 h, the solution was centrifuged at 12,000 rpm for 15 min, and then the absorbance of the supernatant at 540 nm was measured using an M5microplate reader (Molecular Devices, USA). Here, The hemolysis percent was calculated by the following way: Percentage of hemolysis (%) = (As-An)/(Ap-An)×100%, where As, An and Ap is the absorbance of nanoparticles groups, the water, and PBS control group, respectively.

ICP-MS Analysis.

The rabbits were weighed and euthanized after 6 days, 12 days and 30 days. Blood samples, urine and feces were then collected at these different times. The eye, heart, liver, spleen, lung and kidneys were excised and washed thoroughly with phosphate-buffered saline (PBS) to remove residual blood. After the residual water on the organ surface had been removed with filter paper, each sample was weighed and placed in separate beakers and were predigested with aqua regia for Au or HNO_3 for Ag and Cu overnight, followed by digestion with HNO_3 on a hot plate for several h and then cooled and diluted. After that the diluted solution were prepared for quantitative analysis by ICP-MS (PerkinElmer NexION 300X, USA). The samples were placed in separate beakers and were predigested with aqua regia for Au or HNO_3 for Ag and Cu overnight, followed by digestion with HNO_3 on a hot plate for several h and then cooled and diluted. A blank solution and a series of

standard solutions of Au, Ag and Cu diluted in the acid solution were used during the ICP-MS measurements to obtain a standard curve.

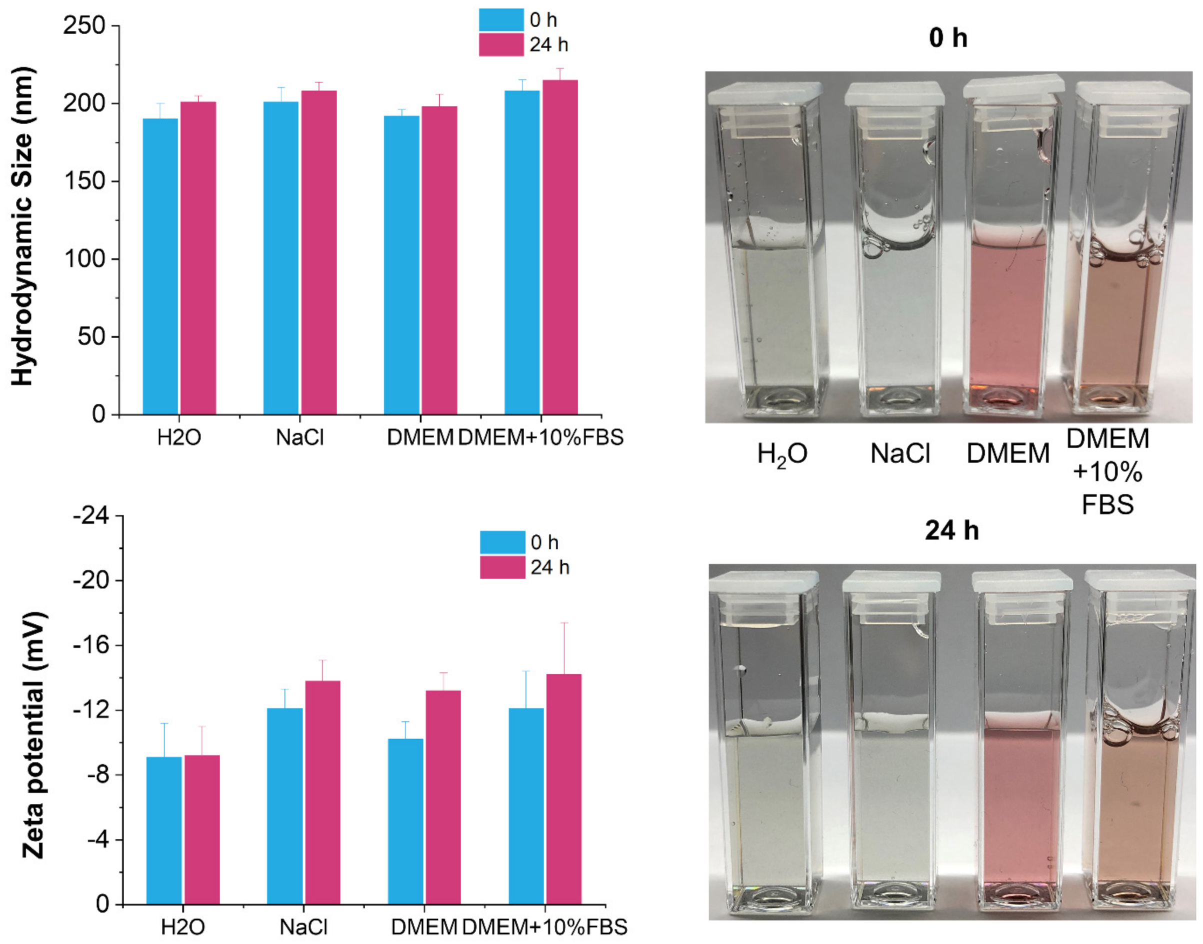

Figure S1. The change of hydrodynamic size and zeta potential of AgAuCu_2O-BS in different physiological solutions (H_2O, 0.9% NaCl, DMEM, and DMEM+10% FBS) for 24h.

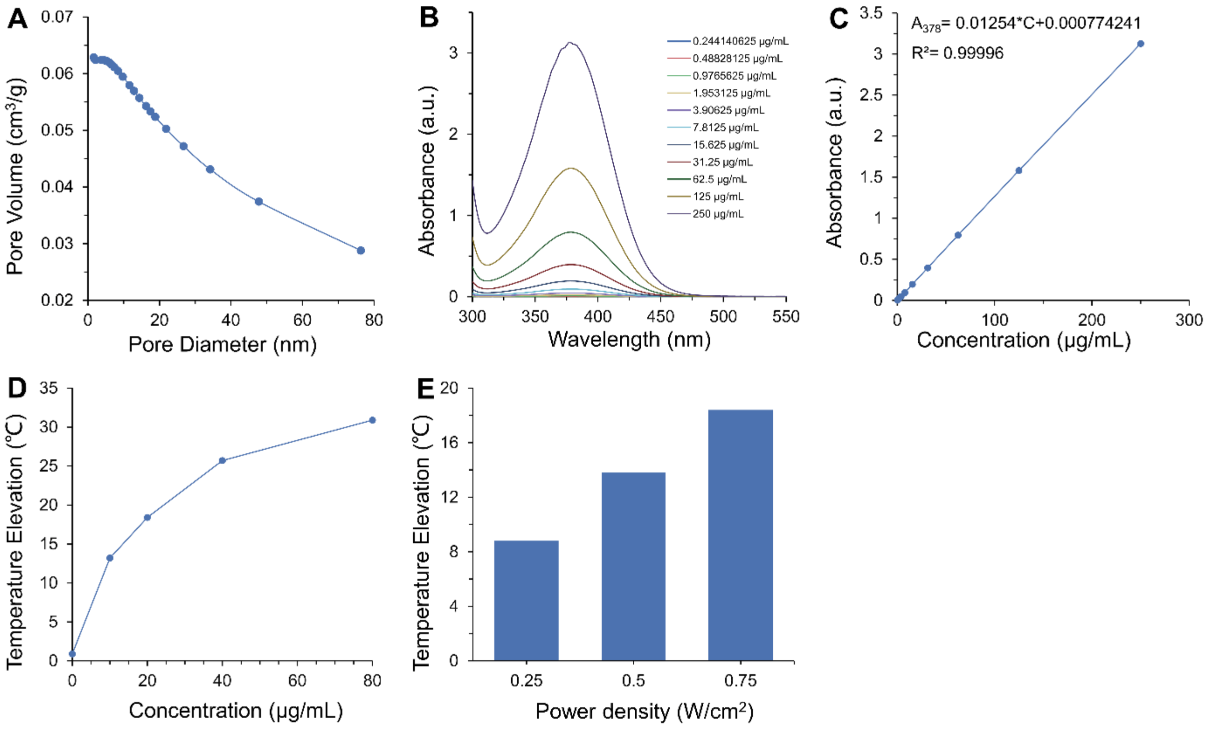

Figure S2. (A) pore volume characterization of $AuAgCu_2O$ NPs. (B) UV-Vis absorption spectrum of bromfenac sodium. (C) Standard curve of the UV absorbance of different concentrations at 378 nm. (D, E) Temperature elevation of different concentrations and power densities under the 808 nm laser for 10 min.

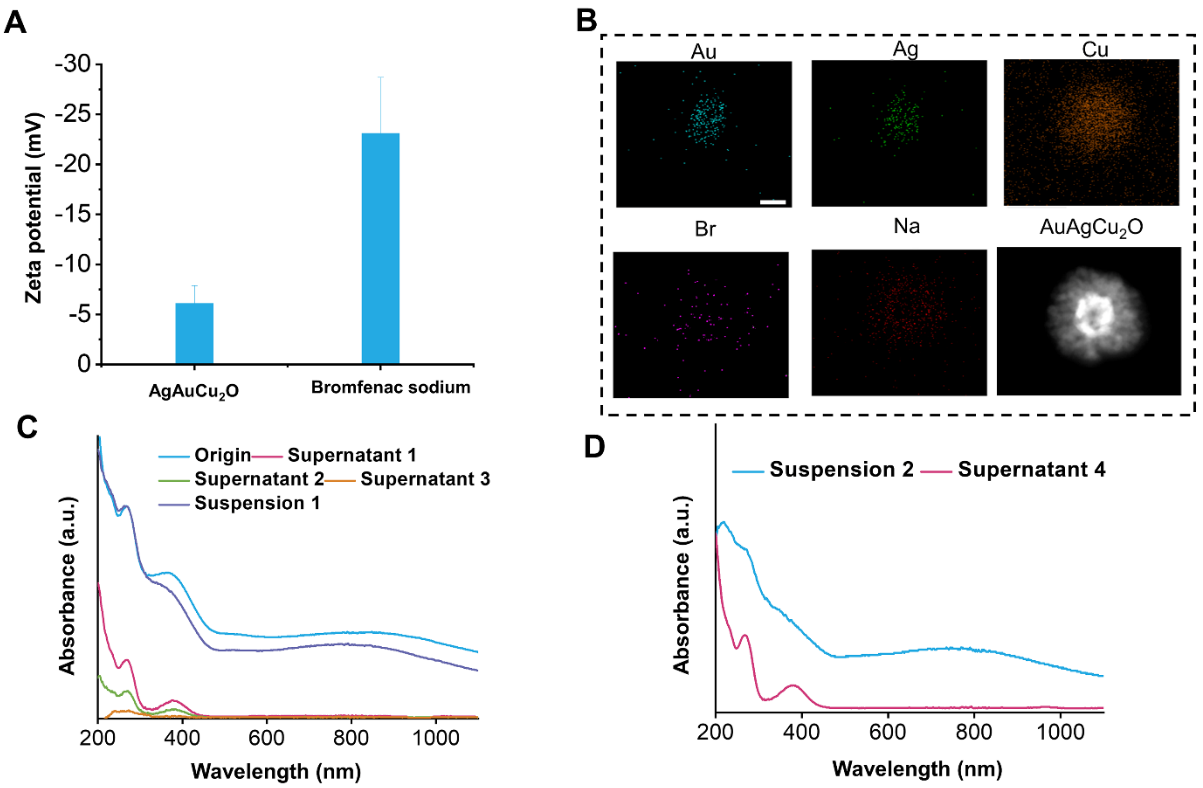

Figure S3. The loading method of drugs. (A) The zeta potential of $AuAgCu_2O$ and bromfenac sodium. (B) The elements distribution mapping of drug-loading nanoparticles ($AuAgCu_2O$-BS). (C) UV−Vis−NIR absorption spectrum of original $AuAgCu_2O$-BS solution (named Origin), the supernatant after centrifugation for three times (titled Supernatant 1, Supernatant 2 and Supernatant 3) and the sediments resuspended solution after the third centrifugation (Suspension 1). (D) UV−Vis−NIR absorption spectrum of the supernatant 4 which was furtherly treated by ultrasonic and the resuspended solution of their precipitate (Suspension 2).

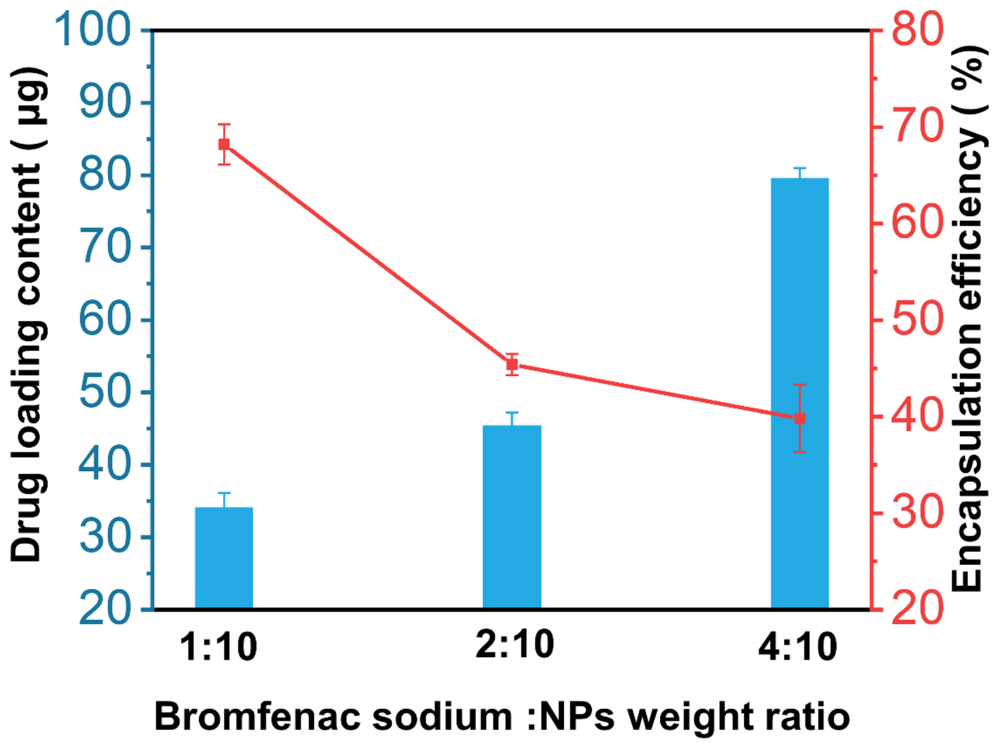

Figure S4. Bromfenac sodium loading content and encapsulation efficiency in $AuAgCu_2O$ nanoparticles at different bromfenac sodium: NPs feeding weight ratio (0.5 mg of NPs, 5 mL).

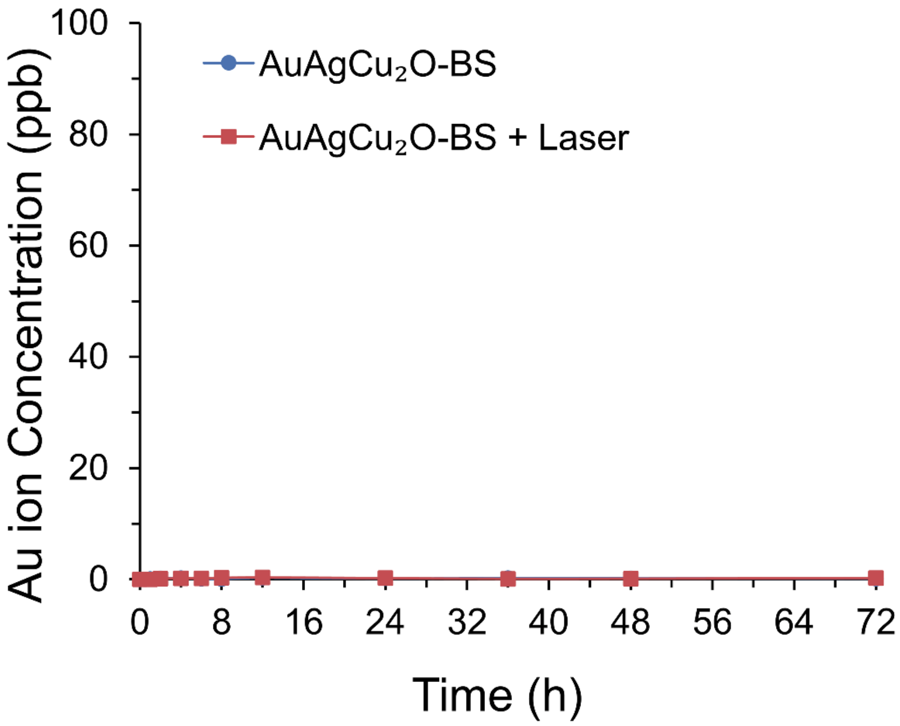

Figure S5. Cumulative amounts of Au ions released from the $AuAgCu_2O$-BS NPs with or without irradiation under the 808 nm laser (0.75 W/cm^2, 10 min).

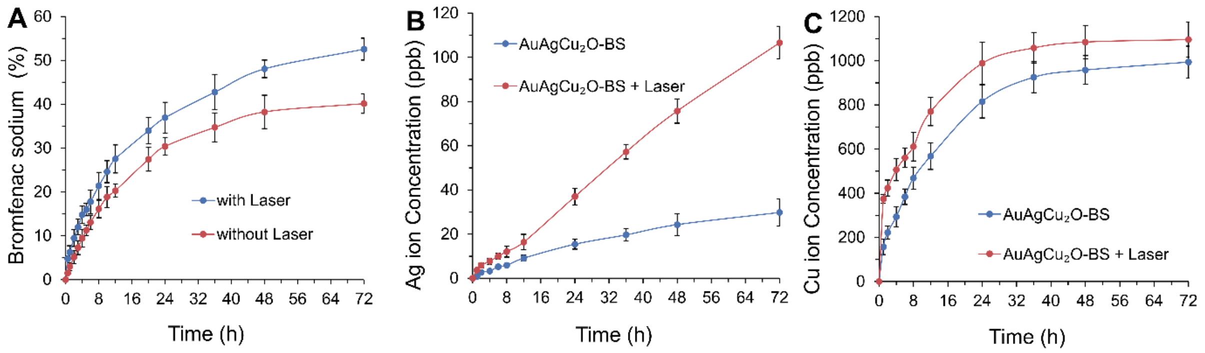

Figure S6. Cumulative amounts of bromfenac sodium, Ag ions, and Cu ions released from the $AuAgCu_2O$-BS NPs with or without irradiation under the 808 nm laser (0.75 W/cm^2, 10 min) in PBS.

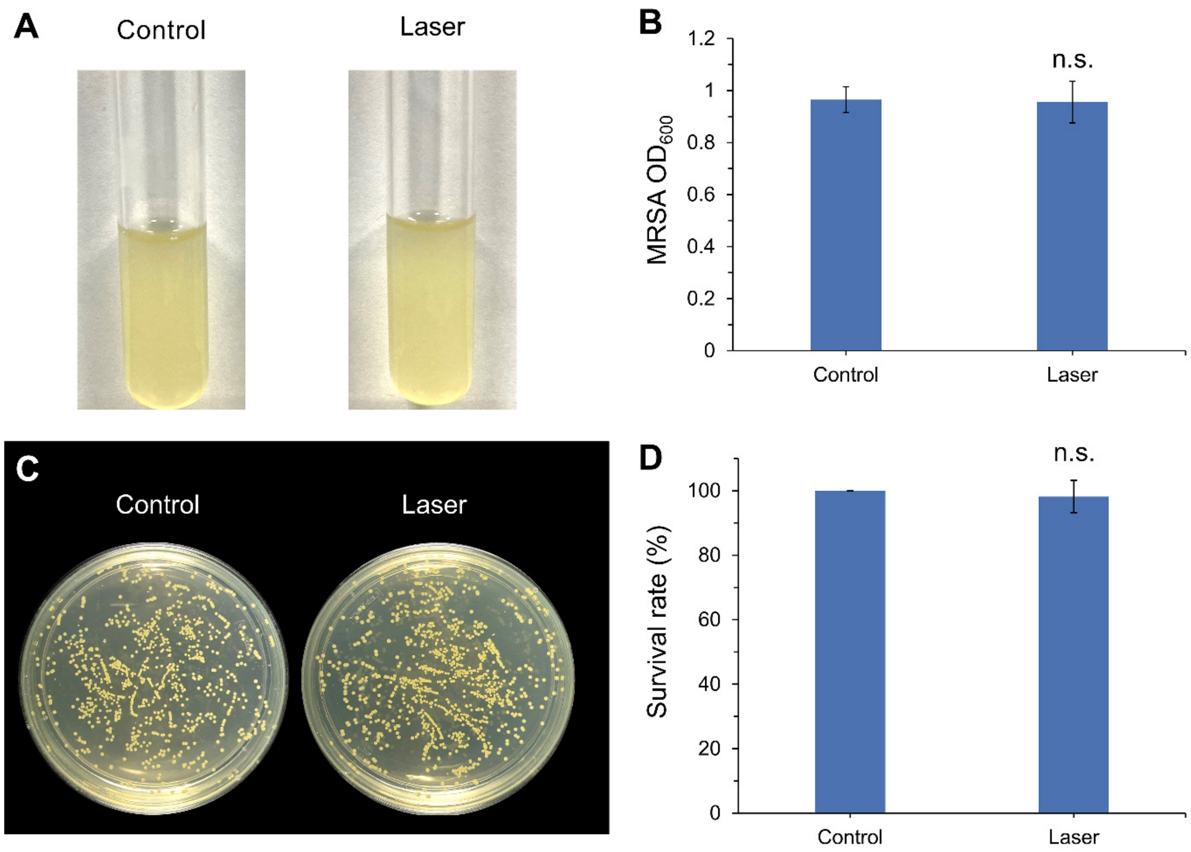

Figure S7. (A) Images and (B) optical density (OD600) of MRSA treated with only laser group (0.75 W/cm^2, 10 min). (C) Plates images and (D) CFU count of MRSA bacterial colonies treated with only laser group (0.75 W/cm^2, 10 min).

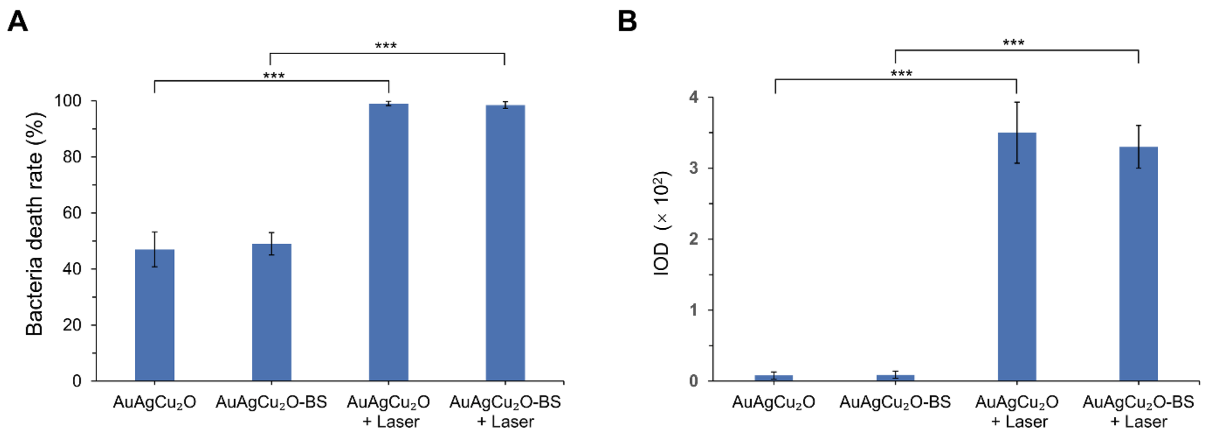

Figure S8. Quantitative analysis of Live/Dead double staining and ROS staining. (A) The bacteria death rate of Live/Dead double staining. (B) integrated optical density (IOD) of ROS staining. Single asterisks (*) indicate $p < 0.001$ compared to the $AuAgCu_2O$- group; pound signs (#) indicate $p < 0.001$ compared to the $AuAgCu_2O$-BS group.

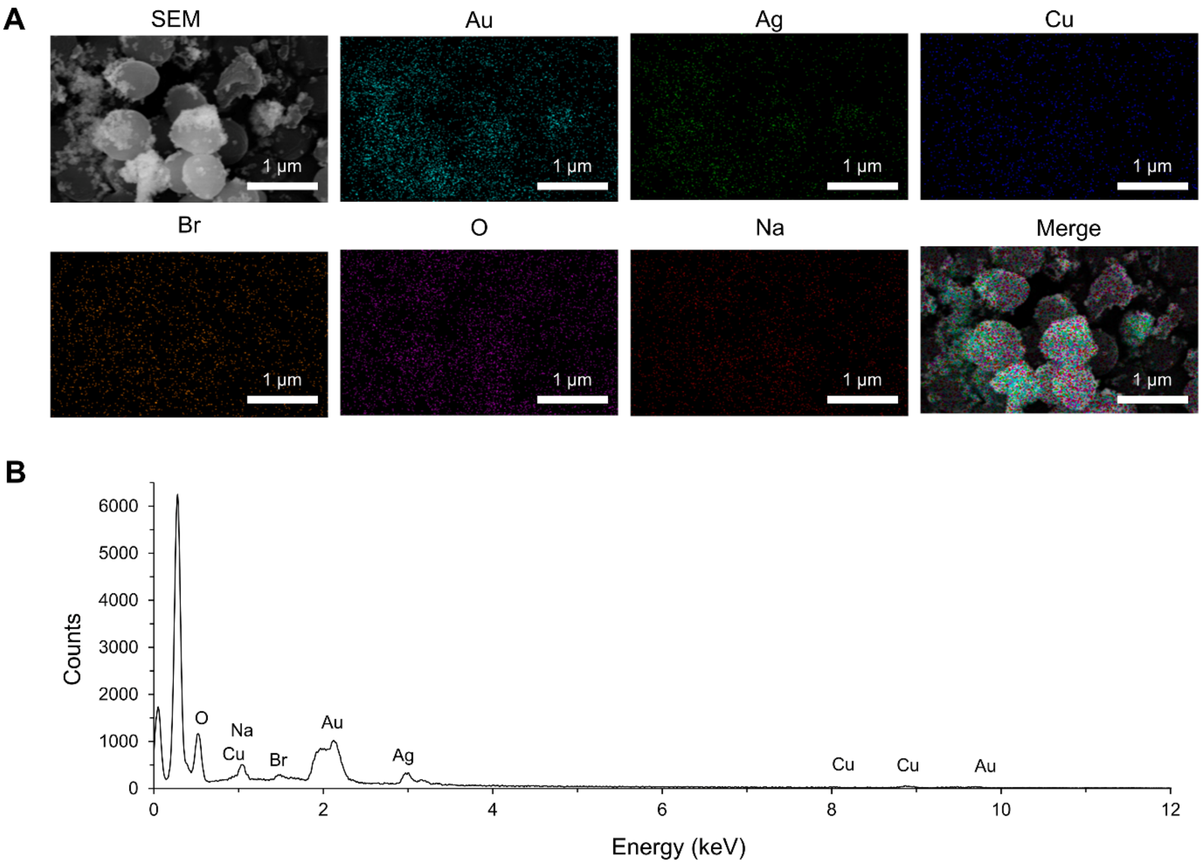

Figure S9. (A) Energy dispersive spectrometer (EDS) element maps and (B) spectrum of MRSA treated with AuAgCu_2O-BS NPs with a laser. SEM indicating the element composition was Au, Ag, Cu, Br, O, and Na.

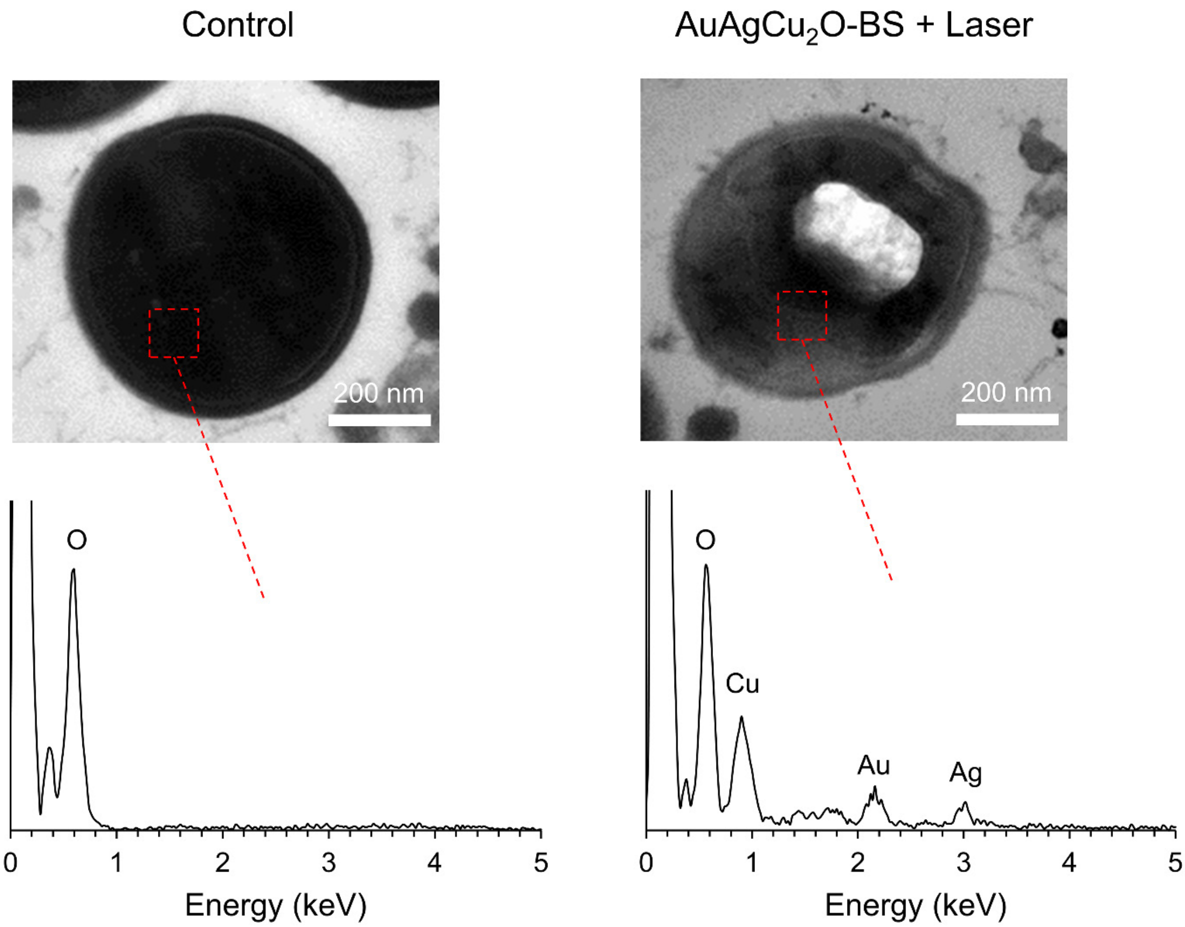

Figure S10. TEM images and corresponding EDS analysis of intracellular sections of different treatment of MRSA.

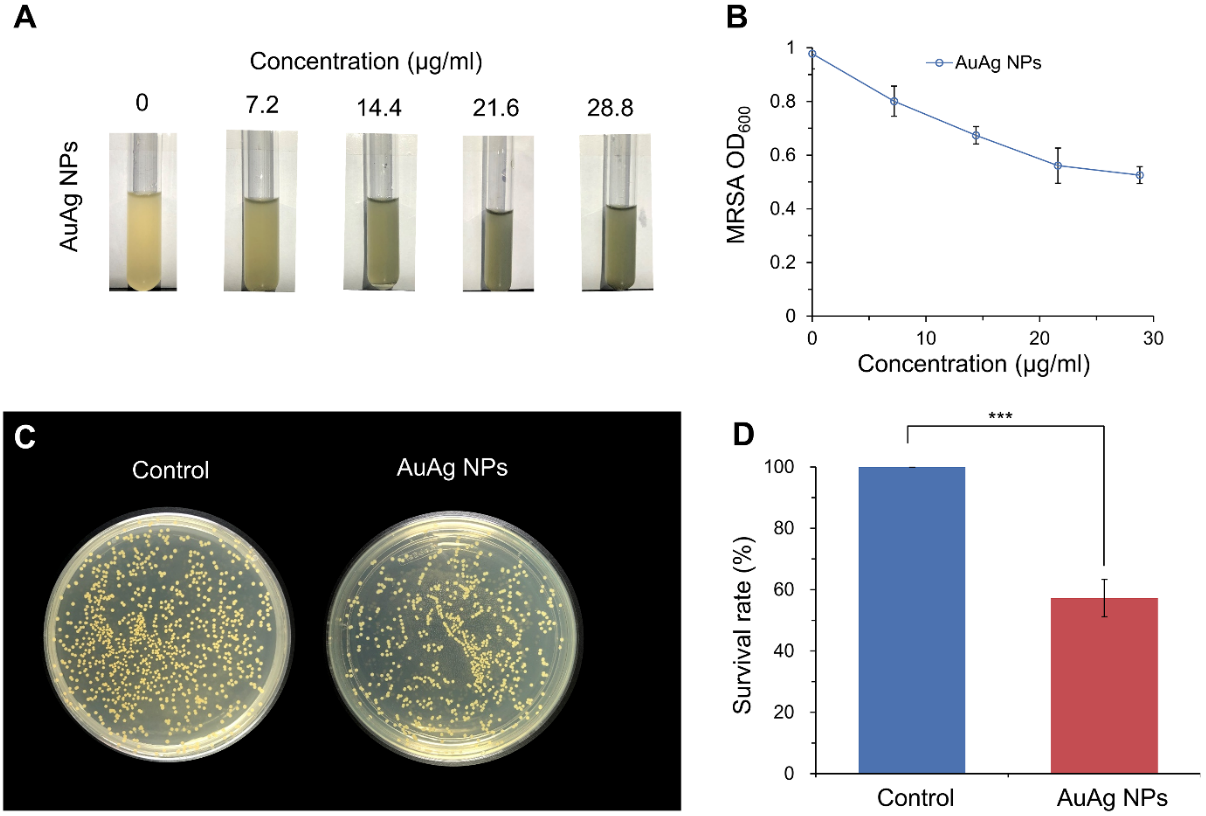

Figure S11. In vitro antibacterial effect study of AuAg NPs. (A) Images and (B) optical density (OD600) of MRSA treated with different concentrations of AuAg NPs. (C) Plates images and (D) CFU count of MRSA bacterial colonies treated with AuAg NPs. (***$p < 0.001$.)

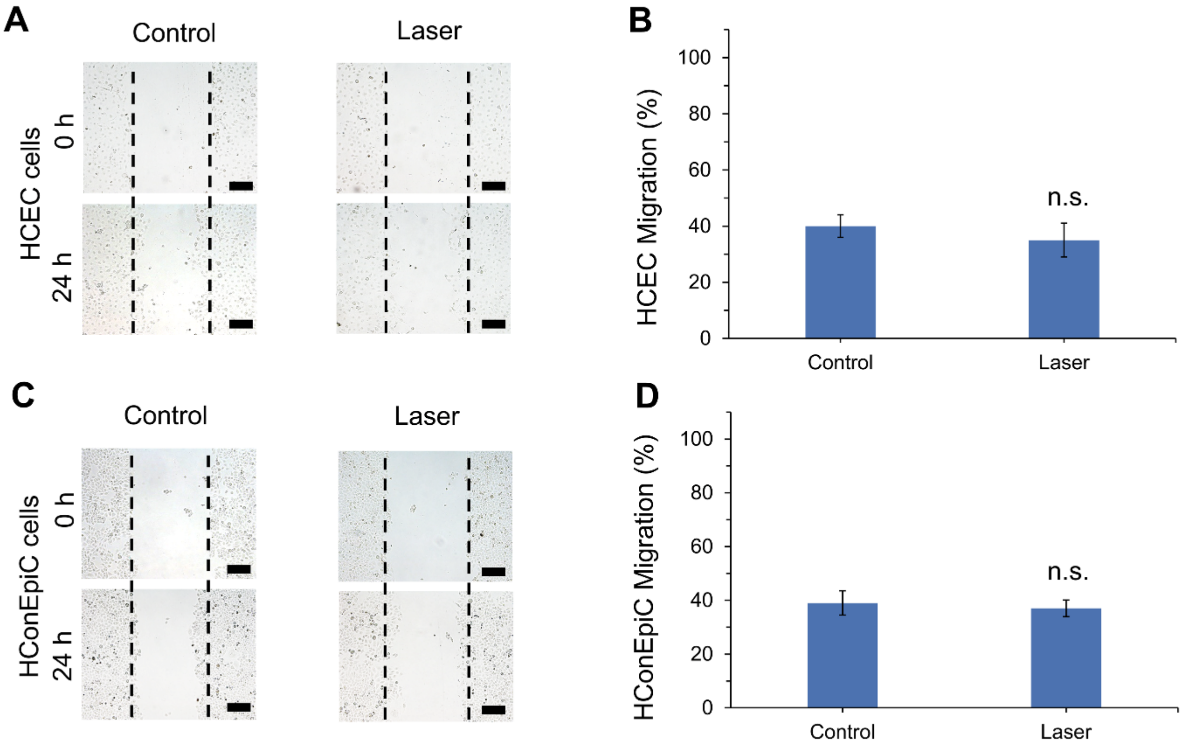

Figure S12. (A, B) Images and quantification of HCEC migration after treatment with only laser group (0.75 W/cm^2, 10 min) , scale bar =400 μm. (C, D) Images and quantification of HConEpic migration after treatment with only laser group (0.75 W/cm^2, 10 min) , scale bar =400 μm. (n.s. $p > 0.05$.)

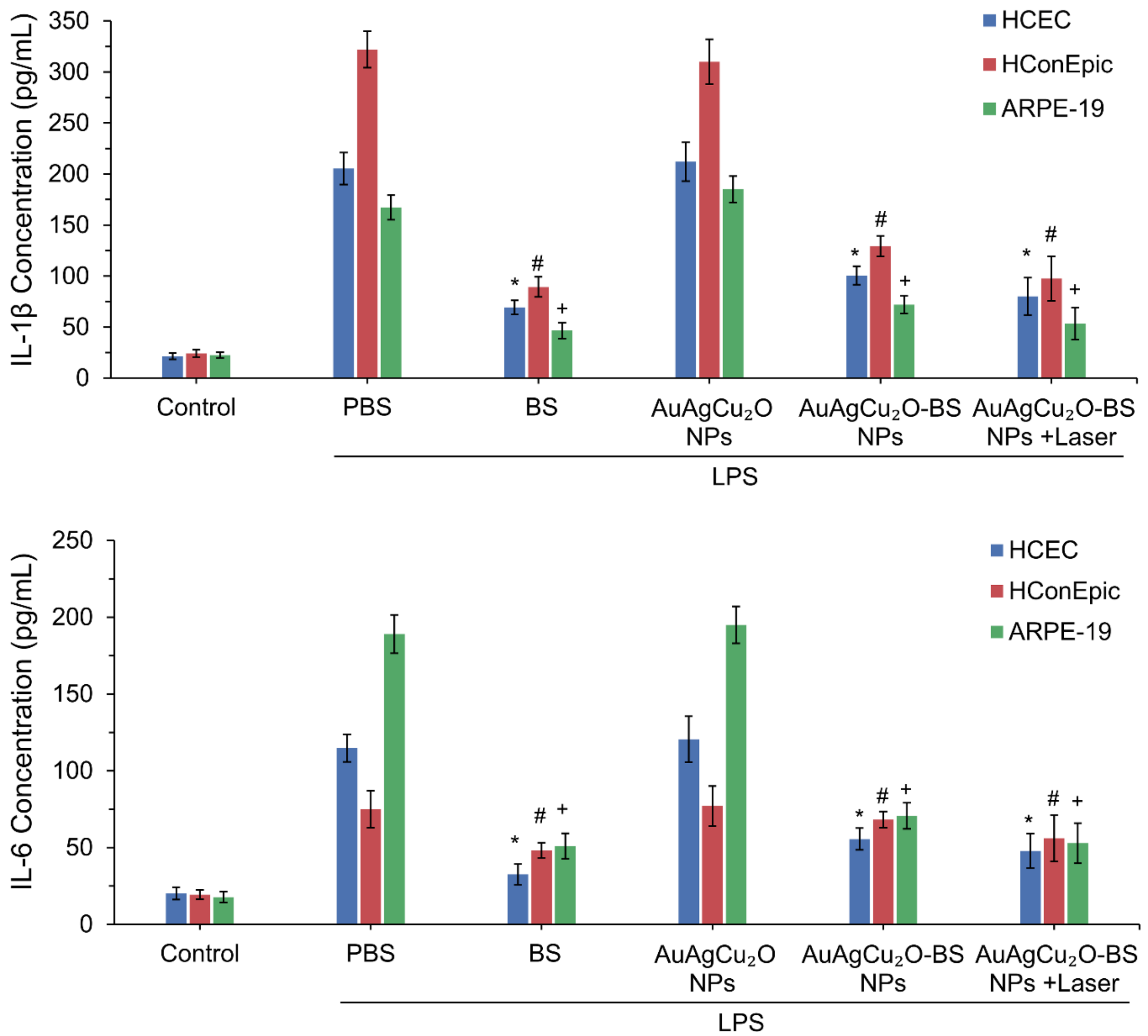

Figure S13. Concentrations of IL-1β and IL-6 in LPS-activated HCEC, HConEpic, and ARPE-19 cells receiving different treatments. Cells without any treatment were used as control. Single asterisks (*) indicate $p < 0.001$ compared to the LPS plus PBS group in HCEC; pound signs (#) indicate $p < 0.001$ compared to the LPS plus PBS group in HConEpic; plus signs (+) indicate $p < 0.001$ compared to the LPS plus PBS group in ARPE -19 cell.

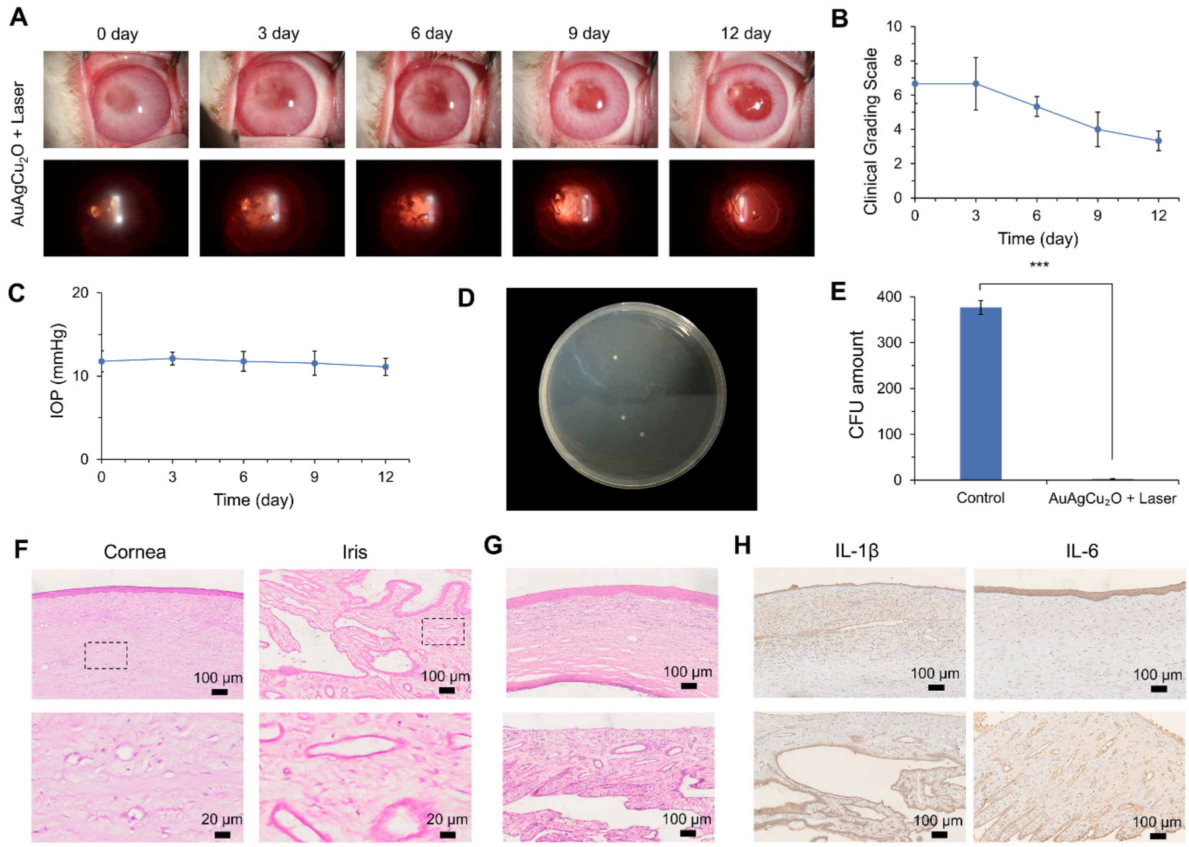

Figure S14. In vivo therapeutic effect analysis of $AuAgCu_2O$ NPs with laser group. (A) Photographs of slit lamp diffuse illumination and retroillumination. (B) The ophthalmological clinical grading scale of endophthalmitis. (C) Measurement of intraocular pressure after treatment. (D) Photographic images of bacterial colonies and (E) counting numbers in aqueous humor after treatment. (F) Gram staining analysis of MRSA in the cornea and iris. The parts framed with the black dotted line were enlarged, respectively. (G) H&E staining analysis of the cornea (top) and iris (bottom). (H) Immunohistochemical staining analysis of IL-1β and IL-6 in the cornea (top) and iris (bottom). (***$p < 0.001$.)

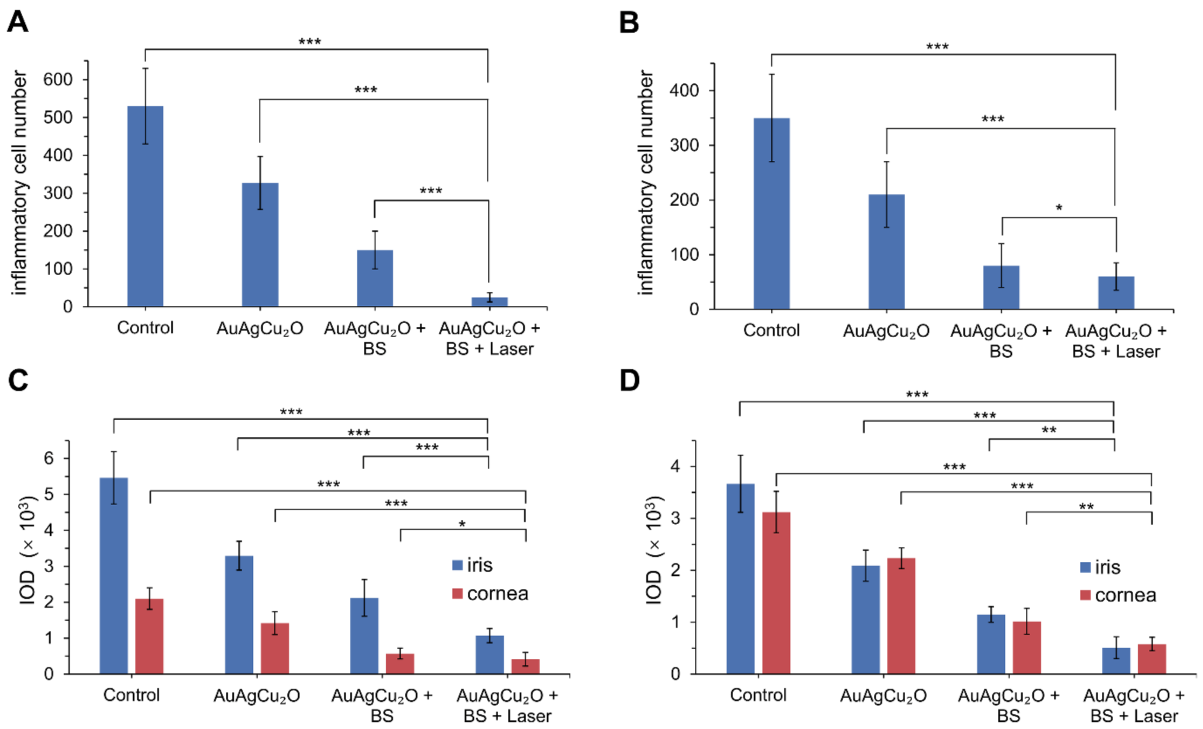

Figure S15. Quantitative analysis of H&E and Immunohistochemical staining. Inflammatory cells number of the cornea (A) and iris (B). Integrated optical density (IOD) of IL-6 (C) and IL-1β (D). Single asterisks (*) indicate $p < 0.001$ compared to the control group.

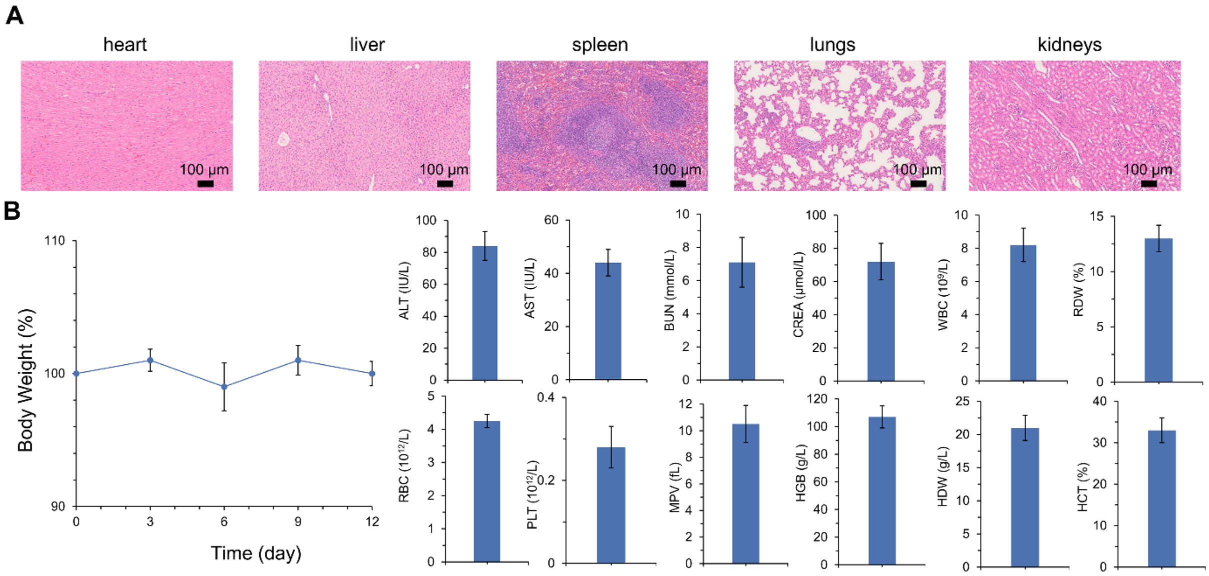

Figure S16. Biosafety Study of $AuAgCu_2O$ NPs with laser group. (A) Toxicological analysis of H&E staining of main visceral organs (heart, liver, spleen, lungs, and kidneys). (B) Changes in body weight and blood biochemistry and blood routine examination analyses. ALT (alanine transferase), AST (aspartate transferase), BUN (blood urea nitrogen), CREA (creatinine), WBC (white blood cells), RDW (red cell distribution width), RBC (red blood cells), PLT (blood platelet), MPV (mean platelet volume), HGB (hemoglobin), HDW (hemoglobin distribution width), and HCT (hematocrit).

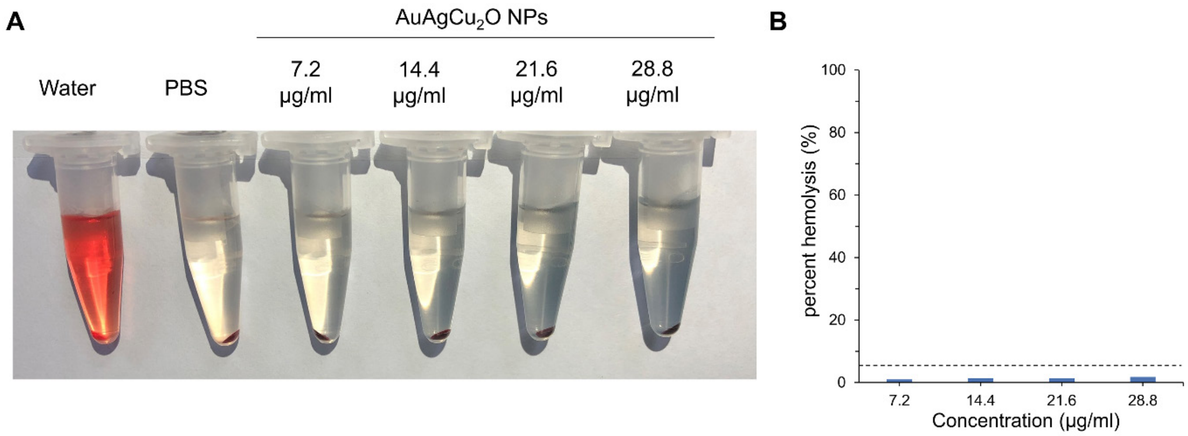

Figure S17. Biocompatibility test. (A-B) Photographs of blood incubated with different concentrations of $AuAgCu_2O$ NPs and corresponding percent hemolysis; water and PBS as the positive and negative controls, respectively.

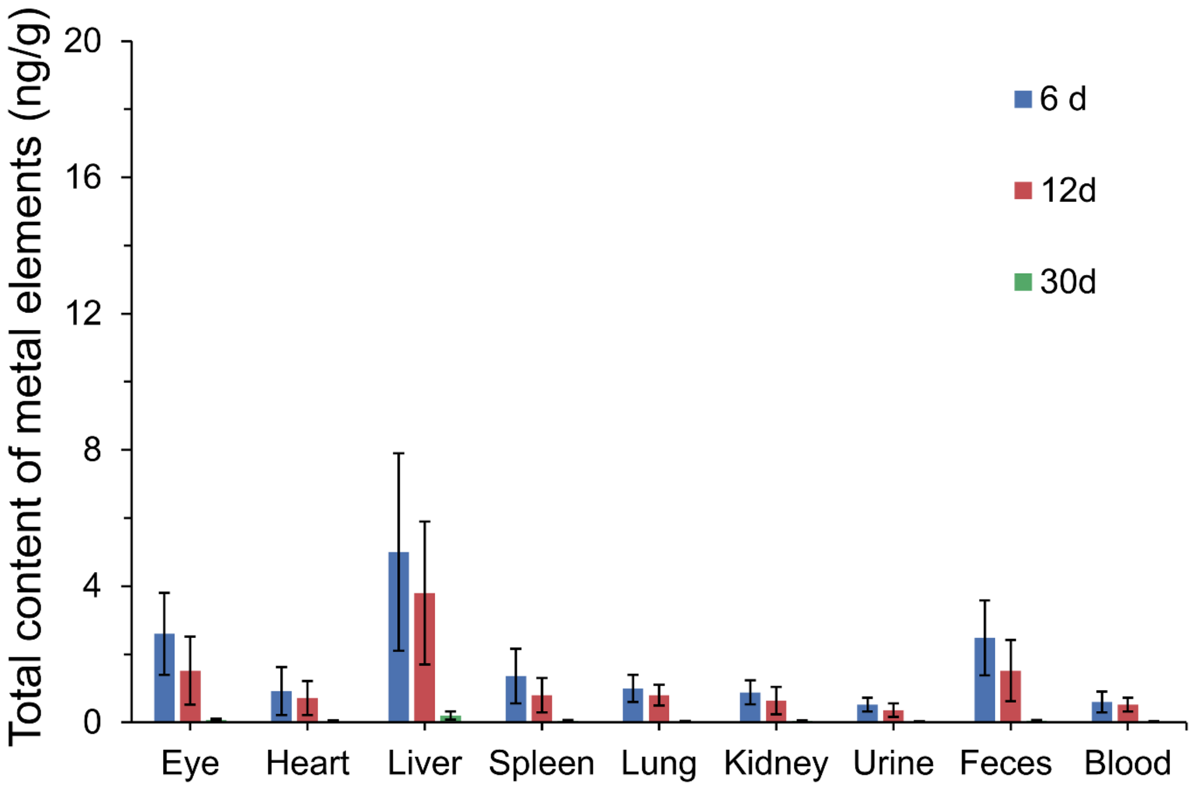

Figure S18. The average content of total metal elements eye, major organs, blood, urine and feces at different time points. All data were deducted by the background value from the blank control.
